# Two activating mutations of *MPL* in triple‐negative myeloproliferative neoplasms

**DOI:** 10.1002/cam4.2387

**Published:** 2019-07-11

**Authors:** Juan Xie, Xiuhua Chen, Feng Gao, Ruixia Hou, Tingting Tian, Yaofang Zhang, Lifang Fan, Jinjun Hu, Guiyang Zhu, Wanfang Yang, Hongwei Wang

**Affiliations:** ^1^ Institute of Hematology The Second Hospital of Shanxi Medical University Taiyuan China; ^2^ Clinical laboratory The First Hospital of Shanxi Medical University Taiyuan China

**Keywords:** molecular pathology, *MPL* mutation, triple-negative myeloproliferative neoplasms

## Abstract

*MPLW515K* or *W515L* mutation plays an important role in the pathogenesis of myeloproliferative neoplasms (MPNs) through signaling molecules of the cytokine receptor axis. Besides *MPLW515K* or *W515L*, more than 30 atypical *MPL* mutations have been reported in patients who are negative for *JAK2V617F, MPLW515K/L*, and *CALR* mutations. Here, we aimed to identify the disease‐causing mutations in the triple‐negative case of ET. We described two *MPL* mutations in patients diagnosed with ET by target sequencing the hotspot mutation region of *MPL* gene. The *MPLA497‐L498ins4* is an insertion mutation detected recurrently in ET patients, and the *MPLW515RQ516E* is a novel double‐point mutation found in an ET patient. Functional studies of *MPLA497‐L498ins4* and *MPLW515RQ516E* revealed that they are gain‐of‐function mutations. Mutants of *MPLA497‐L498ins4* and *MPLW515RQ516E* promoted autonomous proliferation on Ba/F3 cells in the absence of IL‐3. Autonomous activation of TPO‐R without ligand TPO was observed in *MPLA497‐L498ins4* and *MPLW515RQ516E* mutants. Lower percentage of cells in G1 phase and higher percentage of cells in S phase of two atypical *MPL* mutants were detected after culturing without any cytokines. These two atypical *MPL* mutations also presented increase in phosphorylation of signaling proteins including JAK2/STAT, PI3K/AKT, and MAPK/RAS. In summary, the *MPLA497‐L498ins4* and *MPLW515RQ516E* are gain‐of‐function mutations which may be novel driving factors participating in the pathogenesis of triple‐negative MPN.

## 1 INTRODUCTION

Myeloproliferative neoplasms (MPNs) are chronic diseases characterized by clonal hematopoiesis and hyperproliferation of terminally differentiated myeloid cells. Essential thrombocytosis (ET), primary myelofibrosis (PMF), and polycythemia vera (PV) are main diseases of classical BCR‐ABL‐negative MPNs. Most of the MPNs cases are driven by somatic mutations. Around 95% of PV patients, 50% to 60% of ET and 39% to 57% of PMF patients are caused by *JAK2V617F* mutation.^1^ *JAK2V617F* mutation can lead to hyperactivation of the Janus kinase2 (JAK2)/signal transducer and activation of transcription (STAT) signaling pathway.^2^ The second common mutation in ET and PMF is *CALR* exon9 frameshift mutation which has been detected in 25% MPN patients.^3^, ^4^ Almost 80% of *CALR* mutants are *type 1 (52bp deletion)* or *type 2 (5bp insertion)*, which can induce a common frameshift encoding a novel C terminus with high positive charge. These calreticulin mutants exert proliferative function by specifically activating TPO‐R, then bind to the extracellular N‐glycosylation residues to activate the downstream pathway.^5^, ^6^ In addition to *JAK2* and *CALR* mutations, *MPL* mutation is another important molecular marker of ET and PMF.^7^ *MPL* *W515L* or *MPL* *W515K* is the most frequent mutant type, resulting in constitutive activation of TPO‐R and TPO‐R‐JAK2/STAT signal pathways. Besides, other *MPL* mutants have been reported, including *MPLW515A*, *W515S*, *S505N*.^8^, ^9^, ^10^

Mutations of *JAK2*, *CALR*, and *MPL* account for over 90% of MPN cases and are usually mutually exclusive. However, in approximately 15% of essential thrombocytosis (ET) and less than 10% of primary myelofibrosis (PMF), driver mutations are still unknown and these patients are defined as triple‐negative MPN (TN‐MPNs).^11^ Next‐Generation Sequencing (NGS) has identified more than 30 noncanonical *MPL* mutants in TN‐MPNs which are constitutional or acquired somatically. Missense mutations are the major type of atypical *MPL* mutations such as *S204P/F*, *Y591N/D/F*, and so on.^12^, ^13^ Mutations of *MPLW515A*, *W515S*, *W515R*, and *S505N* have been demonstrated to be active and functional,^14^ and *S204P/F* and *Y591N/D/F* mutations are weak gain‐of‐function mutations in MPN. However, more and more functional studies on MPL mutations have indicated that not all of atypical *MPL* mutations are the causative alterations in MPN pathology.^15^ These data attracted our attention that noncanonical *MPL* mutants discovered in patients with TN‐MPNs in clinic need to be further analyzed to verify whether they are real driver mutations in MPN pathology.

In this study, we were intended to identify the driver mutations in triple‐negative cases of ET and PMF. We screened the exon 10 of *MPL* gene to find out other *MPL* mutations relevant for MPN phenotype. Finally, a novel atypical double‐point *MPL* mutation has been identified in our recent study. Since we have described the MPLA497‐L498ins4 without performing the functional analysis in our previous study,^16^ we aimed to assess the functions of these two identified mutations in this study.

## 2 MATERIAL AND METHODS

### 2.1 Patient cohort

Granulocyte DNA samples were collected from 365 triple‐negative patients diagnosed with PV, ET, and PMF who were enrolled in the second hospital of Shanxi medical university (Shanxi, China). The diagnosis of MPN was established according to the 2008 criteria of the World Health Organization. All patients provided informed consent on protocols approved by local ethics committees. Written informed consent was in accordance with the Declaration of Helsinki.

### 2.2 Sanger sequencing

The *MPL* exon10 sequence was amplified by polymerase chain reaction (PCR) and the products were determined by Sanger sequencing, oligo sequences were shown as follows: *MPL* Forward Primer: 5′‐TAGGGGCTGGCTGGATGAG‐3′; *MPL*‐Reverse Primer: 5′‐CTTCGGCTCCACCTGGTCC‐3′. PCR cycling conditions were: 95°C for 2 minutes (Pre‐degeneration), 35 cycles of 94°C for 30 seconds (denaturation), 60°C for 30 seconds (annealing), and 72°C for 30 minutes (extension), final extension in 72°C for 10 minutes, then the products were stored at 4°C.

### 2.3 T‐A clone

PCR product of *MPLW515RQ516E* was connected to the T‐vector pMD^TM^19 (Takara, Japan) by DNA ligation Kit (Takara, Japan). Vectors were transformed to DH5α competent cells. Plasmids were extracted and determined by Sanger sequencing.

### 2.4 Cell lines and cell culture

Interleukin‐3 (IL‐3)‐dependent Murine pro‐B Ba/F3 cells (donated by the Shanghai institute of hematology Ruijin hospital, China) were cultured in RPMI1640 medium (gibico, USA) containing 15% fetal calf serum (gibico, USA) and 10 ng/mL IL‐3 (PerproTech, USA). 293T cells (donated by the Institute of Hematology, Chinese Academy of Medical Sciences, China) were grown in DMEM with 10% fetal calf serum.

### 2.5 Construction of lentiviral expression vectors and Ba/F3 cell models

Full‐length coding sequence (CDS) regions of *MPLWT*, *MPLW515L*, *MPLA497‐L498ins4*, and *MPLW515RQ516E* were obtained from patient samples. Reverse Transcription‐Polymerase Chain Reaction (RT‐PCR) was used to obtain the cDNA sequences, and Primers were designed to amplify the full‐length CDS of *MPL* gene (NM‐005373.2). The sequences of primers for PCR amplification were shown as follows: Forward Primer: 5'‐CCGGAATTCGCCACCATGCCCTCCTGGGCCCTC‐3'; Reverse Primer: 5'‐AAGGAAAAAAGCGGCCGCAGGCTGCTGCCAATAGC‐3'.

PCR cycling conditions were: 95°C for 4 minutes (Pre‐denaturation), 40 cycles of 95°C for 30 seconds (denaturation), 60°C for 30 seconds (annealing), and 72°C for 2 minutes (extension), final extension in 72°C for 10 minutes, then the products were stored at 4°C. After restriction enzyme digestion (Ecor I and Not I, Takara, Japan) and ligation reactions, each sequence was introduced into pCDH1‐MSCV‐MCS1‐T2A‐copGFP lentiviral expression vector (System Biosciences, USA). Then, the lentiviral expression vectors were constructed. The sequence of all constructs was verified using Sanger sequencing. Plasmids were purified by using Endofree Maxi Plasmid kit (Tiangen, China). The lentiviral particles were produced into 293 T cells for 48 hours to later infect IL‐3‐dependent Murine Ba/F3 cells, respectively. Ba/F3 cell models carrying mutations, *MPLWT*, or empty‐vectors were constructed and cultured in RPMI1640 medium containing 15% fetal calf serum and 10 ng/mL IL‐3. Transduction efficiency was analyzed by flow cytometry (Becton Dickinson, USA) after 72‐hour infection. Cells positive for green fluorescent protein (GFP) were sorted by Fluorescence activated Cell Sorting (FACS). The TPO‐R expression rate of each group was analyzed by tagging with anti‐CD110 (Becton Dickinson, USA) by flow cytometry. The positive rate of CD110 and GFP in each group was higher than 90%, indicating that the Ba/F3 cell model expressing *MPL* gene was successfully constructed, and further experiments were carried out.

### 2.6 Site‐directed mutagenesis

The *MPLQ516E* vector was constructed by QuikChange Lightning Site‐Directed Mutagenesis Kit (Agilent Technologies, USA), primers were designed according to the website instruction shown as follows: Forward Primer: 5'‐GTGTGCAGGAAACTCCCCACCTCAGCAGCA‐3'; Reverse Primer: 5'‐TGCTGCTGAGGTGGGAGTTTCCTGCACAC‐3'. PCR conditions were: 95°C (pre‐denaturation) for 2 minutes, 18 cycles of 95°C (denaturation) for 20 seconds, 60°C (annealing) for 10 seconds, and 68°C (extension) for 3 minutes 30 seconds, final extension in 68°C for 5 minutes, then the products were stored at 4°C. PCR product was digested with Dpn‐I (NEB) and then transformed to DH5α competent cells. *MPLQ516E* mutant plasmids were purified using Endofree Maxi Plasmid kit (Tiangen, China) and fully sequenced (BGI, China).

### 2.7 Cell proliferation

For the IL‐3 withdraw test, cells of each group were washed extensively by PBS for three times. Forty thousand Ba/F3 cells per mL (4 × 10^3^/well in 100 μL) of each group were seeded in a 96‐well plate without any cytokine. Cell counts were reflected by the absorbance at 450 nm using Cell Count Kit‐8 (DOJINDO, Japan) after culturing for 0 h, 24 h, 48 h, and 72 h, respectively. Experiments were conducted by Cytation^TM^3 instrument (Bio Tek, USA). To assess the thrombopoietin sensitivity, cells were washed for three times in PBS, and then 40,000 cells per mL were seeded in various concentration of recombinant human TPO (0 ng/mL, 0.001 ng/mL, 0.01 ng/mL, 0.1 ng/mL, 1 ng/mL, 2 ng/mL; PerproTech, USA). After 96 hours, cell counts of each group were detected by Cell Count Kit‐8. All of these experiments were repeated for three times and three replicates were set in each experiment.

### 2.8 Cell cycle analysis

Ba/F3 cells expressing *MPL* mutations or *WT* were stimulated in different concentrations (0 ng/mL, 0.1 ng/mL, 10 ng/mL) of recombinant human TPO for 48 hours without IL‐3 and then 10^6^ cells from each group were collected after being washed by precooling PBS for two times. After fixed in 70% ethanol at 4°C overnight, cells conjugated with 25‐μL PI fluorescence (20X) and 10‐μL RnaseA (50X) were added to 500‐μL buffer (Beyotime, China) and incubated at 37°C for 30 minutes and then measured by flow cytometry. The laser wavelength is 488 nm, and a total of 10^6^ were collected by FSC/SSC. Cells characterized with high Forward scatter/Side scatter (FSC/SSC) profiles were selected and cells presenting low FSC/SSC profiles were regarded as debris or noise. Cells tagged with PI should be distributed in a linear correlation with the Y axis for cell cycle analysis (AUX) signal. Nonlinear related cells were the adherent cells or cell fragments. The histograms of the flow cytometric analysis of the cell cycle of G1, G2, S phase by Modfit software were displayed in Figure S1. The experiment was replicated in triplicates.

### 2.9 Western blotting

Total protein was processed by RIPA buffer (Thermo Fisher Scientific, USA) added with protease and phosphatase inhibitor mixture tablets (Thermo Fisher Scientific, USA). Each group of Ba/F3 cells was starved without IL‐3 for 48 hours, and then the protein was extracted. Signaling studies were performed on Ba/F3 cell lines by western blot analysis of JAK2 (Tyr1007/1008), STAT1 (Tyr701), STAT3 (Tyr705), STAT5 (Tyr 694), p44/p42 MAPK (Erk1/2)(Thr202/Tyr 204), and AKT (Thr 308) and of these different phosphorylated proteins, β‐actin (Abcam, UK) was considered as the house keeping protein of all groups. Western blotting was performed by Simple Western Kit (Protein Simple, USA). Five microgram of total protein from each group was loaded to WES 25‐well plates for separation (Protein Simple, USA). The antibody and phosphorylated antibody of JAK2, STAT1, STAT3, STAT5, AKT, p44/p42 MAPK (Erk1/2) were used as primary antibodies. The HRP‐conjugated anti‐rabbit secondary antibody was applied according to the instructions of simple western kit (Protein Simple, USA). The dilution ratio was 1:100 in antibodies of JAK2, STAT1, STAT3, STAT5, AKT, p44/p42 MAPK (Erk1/2), p‐STAT1, p‐STAT3, p‐STAT5, p‐p44/p42 MAPK (Erk1/2), and β‐actin, and antibodies of p‐JAK2 and p‐AKT were diluted in 1:50. The relative amount of each protein was analyzed through the areas under peaks from the chemiluminescence chromatograms by the Compass for SW software (Protein Simple). The western blot analysis was repeated twice. The catalog number of regents and kit for Western blot (WB) are provided in Table S1.

### 2.10 Statistical analysis

Results exhibited in figures, including the cell counts and the percentage of cell cycle of G1, G2 and S phases, were illustrated as the mean ± standard deviation (SD) of three independent trials. Statistical analysis was performed by one‐way ANOVA. After the analysis of Shapiro‐Wilk test and variance homogeneity test, data obeying normal distribution and homogeneous variance were tested by LSD test, and Tamhane's test was used for data with inhomogeneity of variance. The Kruskal‐Wallis test is executed for non‐normal distribution data. Statistical analysis was performed using SPSS19.0 software (SPSS Inc, Chicago). For all analyses, the p values were two‐tailed and *P* < 0.05 was considered statistically significant.

## 3 RESULTS

### 3.1 Identification of two atypical MPL mutants

**Figure 1:**
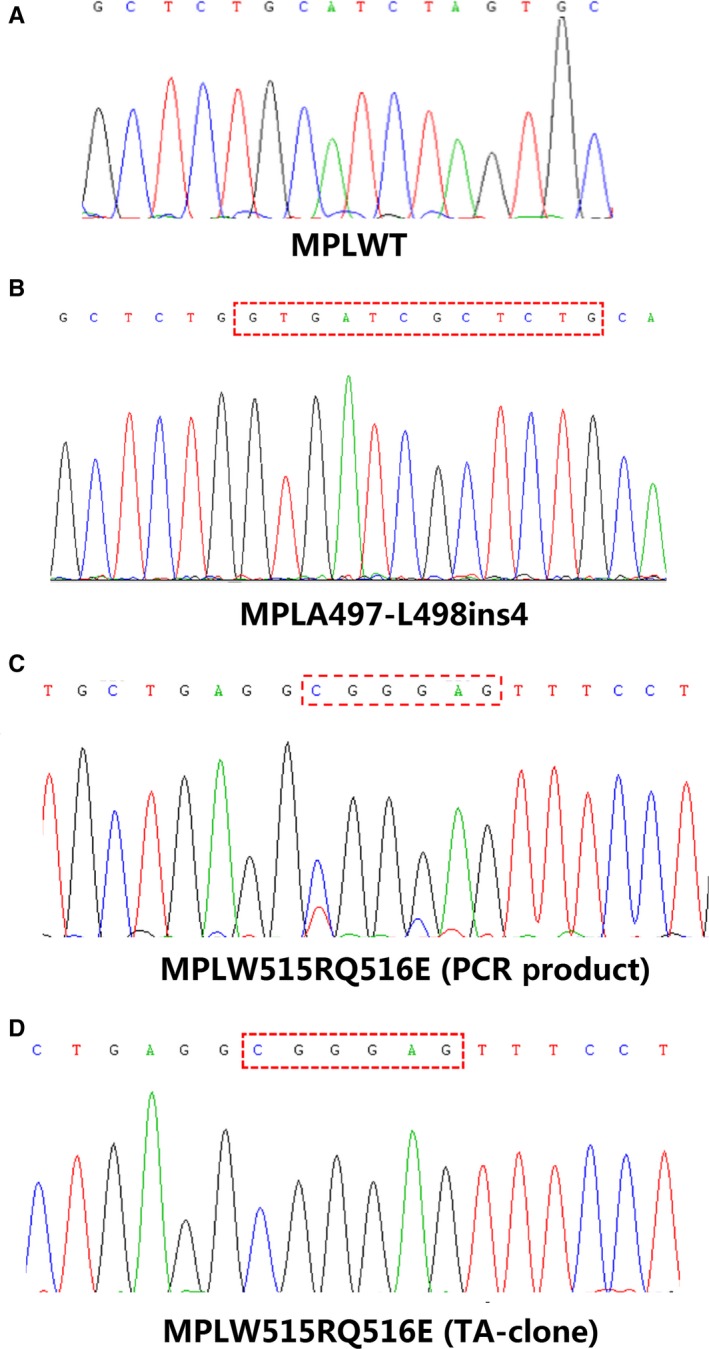
Atypical *MPL* mutations identified in triple‐negative myeloproliferative neoplasms. (A) *MPL wide type (WT)* control sequence; (B) *MPLA497‐L498ins4* mutation identified in another case of essential thrombocytosis. (C) PCR‐product sequence of *MPLW515RQ516E* mutation; (D) TA‐clone identified two mutant points of *W515R and Q516E* occurred in the same DNA strand

We screened the hotspot mutation region of *MPL* gene (exon 10) in 365 triple‐negative patients in our hospital from 2010 to 2017 in Shanxi province, China and identified two atypical *MPL* mutants, *MPLA497‐L498ins4* (Figure 1B) and *MPLW515RQ516E* (Figure 1C). These three MPN patients were negative for *JAK2*, *CALR*, and other atypical *MPL* mutations, such as *S204P* or *Y591D*. In our previous study, we have described *MPLA497‐L498ins4* mutation in one patient with ET. In the current study, we detected this mutation in another triple‐negative ET patient again. The mutation frequency of *MPLA497‐L498ins4* in triple negative MPN is around 0.548% in our center. *MPLA497‐L498ins4* locates at the site of 497 amino acid, which inserts 12bp coding for LVIA amino acid. We have demonstrated that the *MPLA497‐L498ins4* was a somatic mutant in our previous study. Another atypical mutation of *MPL* was found only in a female ET patient, which caused thymidine to cytosine and cytosine to guanine substitution at nucleotides 1543 and 1546, respectively, and resulted in a Tryptophan to Arginine and a Glutamine to Glutamate substitution at codons 515 (*W515R*) and 516 (*Q516E*). To determine whether two mutation sites were located in the same clone, we performed the TA‐clone experiment and found that the *W515R* and *Q516E* mutations were in the same DNA strand (Figure 1D), then we named it as *W515RQ516E*.

### 3.2 Two atypical *MPL* mutations promote cytokine‐independent proliferation on Ba/F3 cells

**Figure 2:**
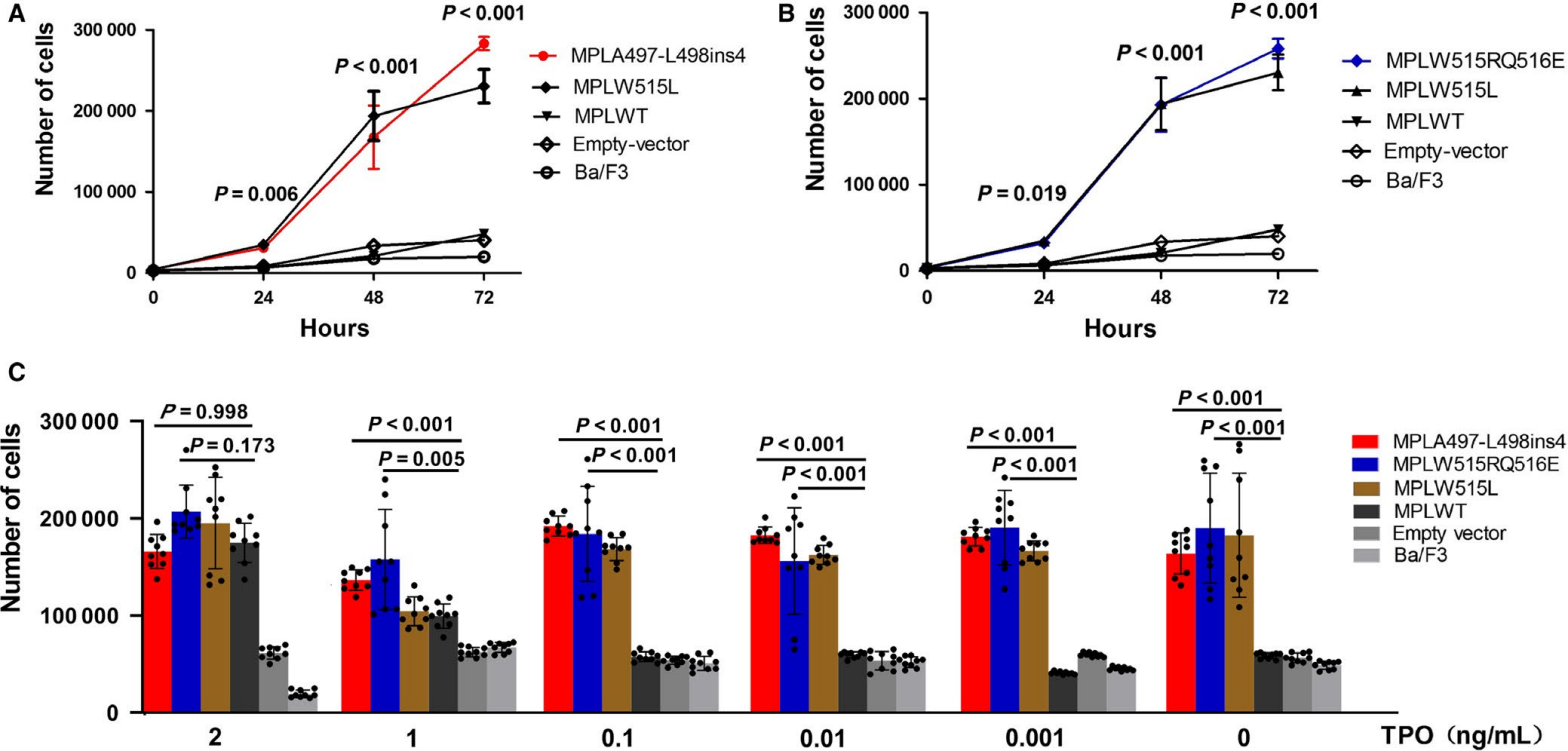
*MPLA497‐L498ins4* and *MPLW515RQ516E* mutations conferred growth advantages in vitro. *MPLA497‐L498ins4* and *MPLW515RQ516E, MPLW515L, MPLWT,* empty vector, and Ba/F3 cells were washed extensively and cultured in the absence of cytokines at an initial concentration of 4*10^4^ cell per mL. Shown were the means ± standard deviation (SD) of three independent experiments and error bars represented SD. (A‐B) The *MPLA497‐L498ins4* (A) and *MPLW515RQ516E* (B) mutants exhibited obvious independent proliferation compared with MPL wild‐type group**.** Cell counts were detected by CCK‐8 at 0 h, 24 h, 48 h, 72 h, respectively. Nine replicates from three independent experiments were exhibited on figures and analyzed by ANOVA. (C) The *MPLA497‐L498ins4* and *MPLW515RQ516E* mutants presented a hypersensitivity of TPO in low concentration of TPO and can maintain auto‐proliferation even in the absence of TPO. Each group of cells were cultured at an initial concentration of 4*10^4^ cell per mL for 4 days with various concentrations of TPO (0 ng/mL, 0.001 ng/mL, 0.01 ng/mL, 0.1 ng/mL, 1 ng/mL, and 2 ng/mL). Shown were nine replicates from three independent experiments. One‐way ANOVA analysis was used for data following normal distribution, and otherwise, the Kruskal‐Wallis test was performed to determine at 5% significance level

To determine the function of two novel *MPL* mutant*s*, we constructed Ba/F3 cell models expressing these mutations, respectively, to investigate their ability of autonomous proliferation on Ba/F3 cells. In cell culture assay, *MPL* mutants of *A497‐L498ins4* and *W515RQ516E* both conferred cytokine‐independent growth on Ba/F3 cells (Figure 2A,B). In the absence of IL‐3, we observed that *MPLA497‐L498ins4* and *W515RQ516E* exhibited marked growth on Ba/F3 cells, similar to the *W515L*, which was used as a positive control, whereas *MPLWT*, empty vector, and un‐transfected Ba/F3 cells did not grow optimally. The thrombopoietin (TPO), is the special ligand to TPO‐R. As *MPLW515L* mutation can cause striking hypersensitivity to its ligand, we considered whether two novel *MPL* mutations were able to induce the similar effect. Thus, we tested the viability of two novel mutants in the dilution series of TPO. After cultured at the low level or in the absence of TPO for 96 hours, cells expressing *MPLA497‐L498ins4* and *MPLW515RQ516E* mutations showed high sensitivity to TPO or even grew autonomously compared to *MPLWT*, empty vector, and un‐transfected Ba/F3 cells (Figure 2C). However, there was no difference between Ba/F3 cells carrying *MPL* mutants and *MPLWT* at the high TPO concentration (2 ng/mL).

These results indicated that *MPLA497‐L498ins4* and *MPLW515RQ516E* mutations are gain‐of‐function mutations and can cause the autonomous activation of TPO‐R without ligand TPO.

### 3.3 The single *MPLQ516E* mutation exerted weak auto‐proliferation on Ba/F3 cells and resulted in the autonomous activation without TPO ligand

**Figure 3:**
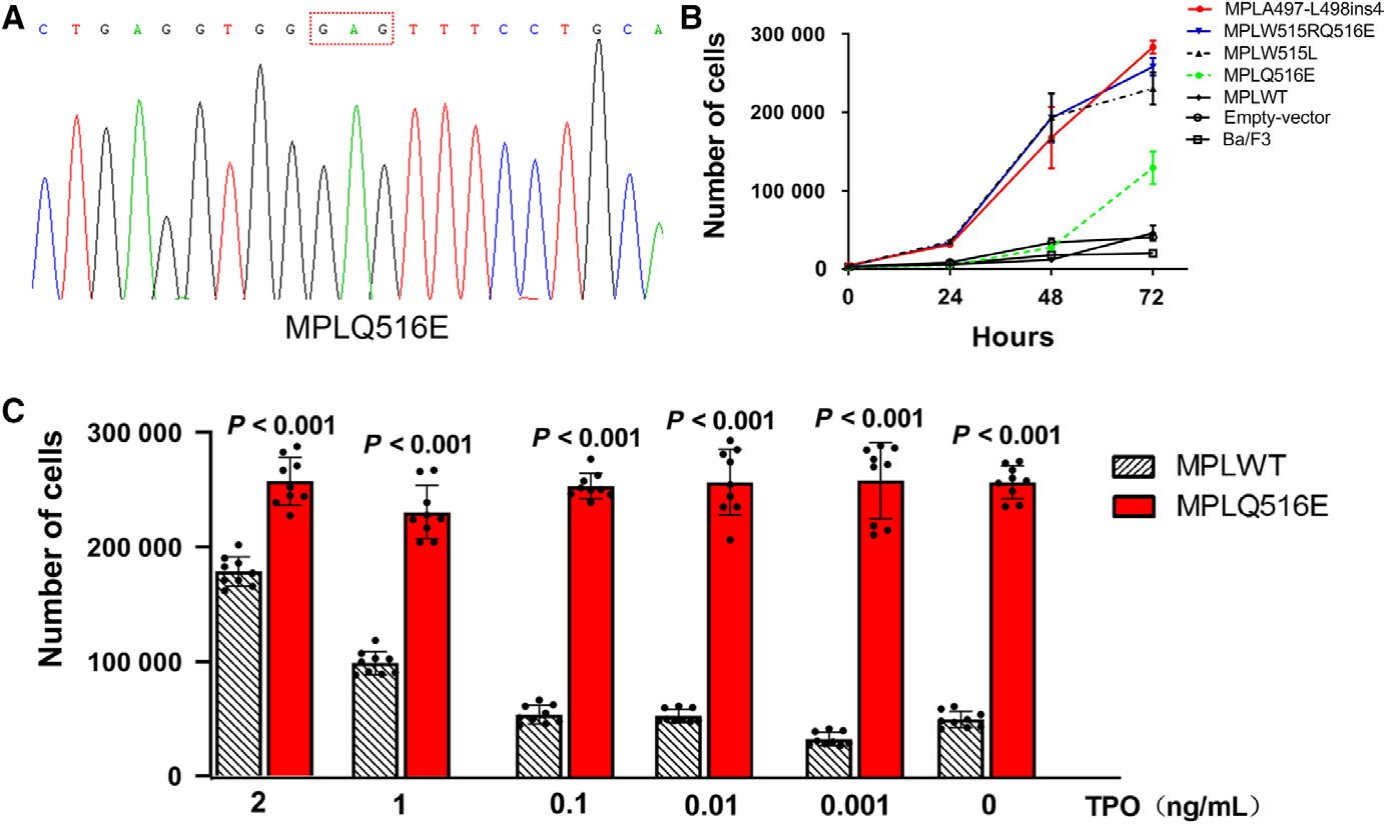
The *MPLQ516E* is a weak activating mutation. (A) The *MPLQ516E* mutant vector was generated using site‐directed mutagenesis, and fully sequenced by Sanger sequencing. (B) *MPLQ516E* mutant promoted the cytokine‐independent growth on Ba/F3 cells compared to *MPLWT but* which was much weaker than *MPLA497‐L498ins*, *MPLW515RQ516E,* and *MPLW515L* mutation. Showing in figures were means ± standard deviation (SD) of three independent experiments done in triplicate. Error bars represented SD. Data were analyzed by ANOVA. (C) The *MPLQ516E* mutation shown a hypersensitivity of TPO and grew faster than the *MPLWT* cells in each TPO condition. Experiments were repeated for three times. The data were shown as the average of three biological replicates performed in triplicate and error bars represented SD. Data were analyzed by ANOVA

As the *MPLW515RQ516E* is an activating mutation leading to cytokine‐independent growth on Ba/F3 cells, we wondered whether the single‐site mutation of *MPLQ516E* played a role in the process. To further identify the function of *Q516E* mutation, we substituted the codon of “CAG” by “GAG” using the site‐directed mutagenesis kit (Figure 3A), and then the lentiviral vector of *MPLQ516E* was generated. Cells expressing *MPLQ516E* stably were constructed after 72‐hour lentiviral infection. It was interesting that the *Q516E* mutation alone survived much better than *MPLWT* (*P* < 0.001) and induced an autonomous proliferation effect on cells after withdrawing the IL‐3 cytokine. However, it is noticeable that the proliferation efficiency of single mutation *Q516E* was much lower than that of *A497‐L498ins4* (*P* < 0.001), *W515RQ516E* (*P* < 0.001), and *MPLW515L* mutation (*P* < 0.001) (Figure 3B and Table 1).

**Table 1:** The *P*‐value of IL‐3 withdraw test between mutants of *MPLA497‐L498ins4, MPLW515RQ516E, MPLW515L, and MPLQ516E* mutation

	Vs *MPLQ516E*			
	0 h	24 h	48 h	72 h
*MPLA497‐L498ins4*	0.344	<0.001	<0.001	<0.001
*MPLW515RQ516E*	0.12	<0.001	<0.001	<0.001
*MPLW515L*	0.23	<0.001	0.001	<0.001
*MPLWT*	0.61	0.155	<0.001	<0.001
Empty vector	0.153	<0.001	<0.001	<0.001
Ba/F3	0.836	0.261	<0.001	<0.001

Furthermore, the viability of *MPLQ516E* under various concentrations of TPO was also tested. After 96 hours, numbers of cell were determined. Cells stably expressing the mutant *Q516E* exhibited increased proliferation in response to TPO, particularly at low concentration, when compared to cells expressing *MPLWT* (Figure 3C).

### 3.4 *MPLA497‐L498ins4* and *MPLW515RQ516E* promoted the G1/S‐phase transition on Ba/F3 cells

**Figure 4:**
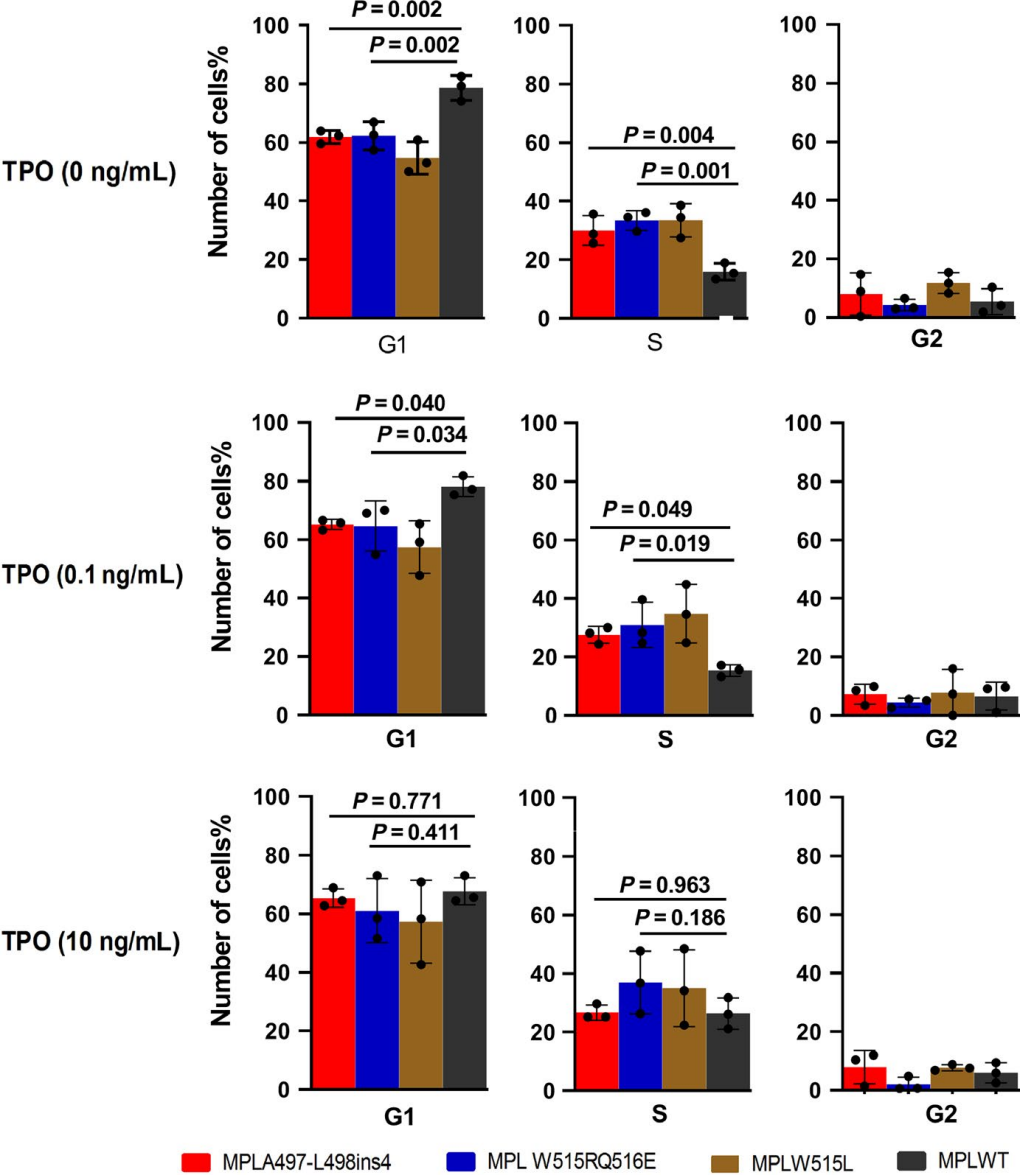
Cell cycle analysis on *MPLA497‐L498ins4* and *MPLW515RQ516E* mutants. *MPLA497‐L498ins4* and *MPLW515RQ516E*, *MPLW515L*, *MPLWT* were cultured for 48 h with various concentrations of TPO (0 ng/mL, 0.1 ng/mL, 10 ng/mL), and the percentage of G1, G2, S phases cells was analyzed by flow cytometry using the Propidium iodide staining. The percentage of G1, G2, S phases on *MPL* mutation groups was compared to *MPL* wild‐type group. Shown results were means ± standard deviation (SD) of three independent experiments and error bars represented in figures were SD. Data were analyzed by ANOVA

We performed the cell cycle analysis to confirm at which step of the cell cycle the *MPLA497‐L498ins4* and *W515RQ516E* mutations conferred a proliferative advantage. Cells were stimulated in various concentrations of TPO for 48 hours, and then the percentage of G1, G2, and S was analyzed. The data showed that Ba/F3 cells expressing *MPL* mutation*s* reached a low percentage of G1 phase and high percentage of S phase (Figure 4). After withdrawing both IL‐3 and TPO for 48 hours, there were still high percentage of cells entering to S phase in *MPLA497‐L498ins4* and *MPLW515RQ516E* groups (30% and 33.4%, respectively) while the *MPLWT* group was in a state of stagnation of G1 phase (78.6% in G1 phase and only 15% in S phase). Under low concentration of TPO (0.1 ng/mL), 27.7% *MPLA497‐L498ins4* cells, 30.9% *MPLW515RQ516E* cells, and 34.8% *MPLW515L* cells were in S phase, whereas only 15.3% of *MPLWT* cells were in S phase. In contrast, with high concentration of TPO (10 ng/mL), all of four groups reached a high percentage of S phase (26.6%, 36.9%, 34.9%, and 26.3%, respectively). Compared with previous studies that atypical *MPL A506T, L510P,* and *A519T* mutations rarely promote a G1/S‐phase transition on cells,^17^ our study further clarified that two atypical *MPL* mutations promoted cells to enter into S phase regardless of the concentration of TPO, and synthesize DNA actively.

### 3.5 Effect of *MPLA497‐L498ins4* and *MPLW515RQ516E* mutations on signal transduction

**Figure 5:**
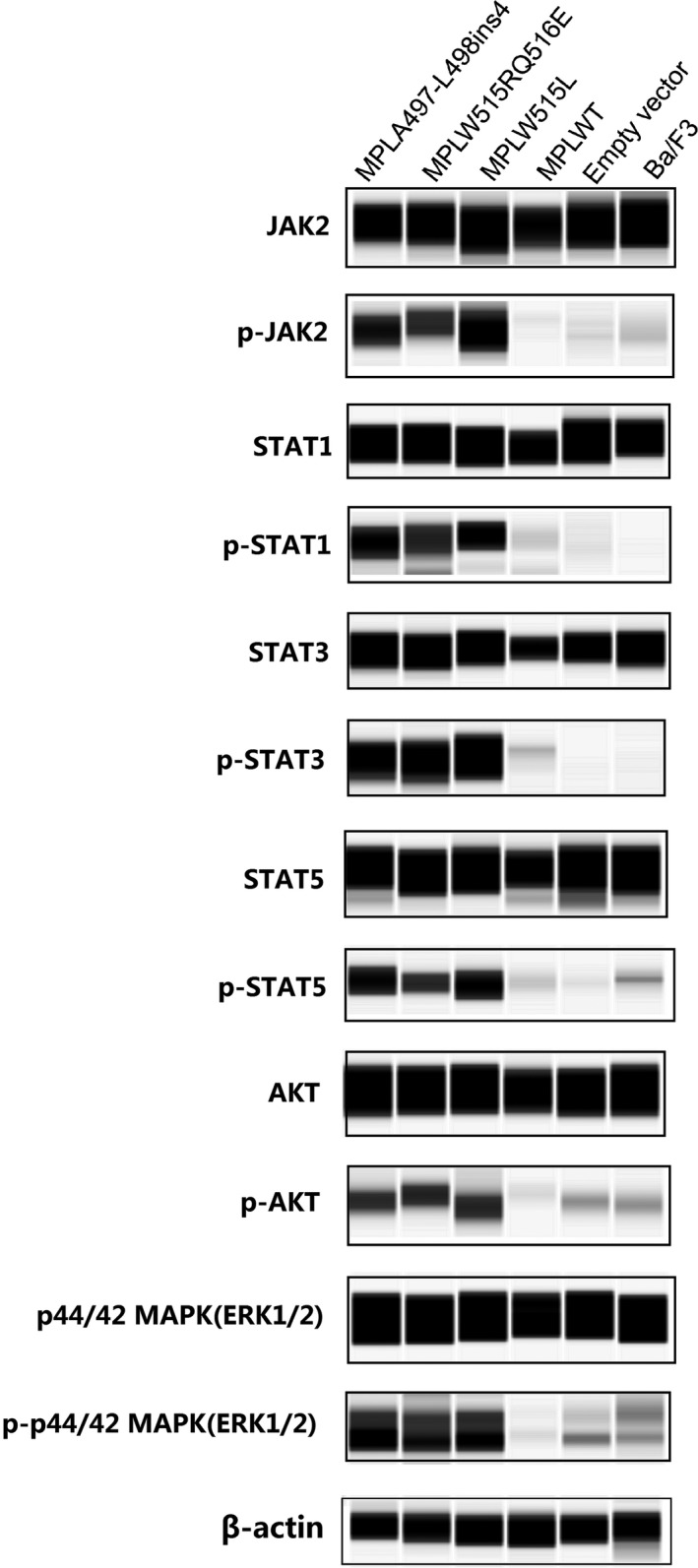
Signal pathway activation analysis of *MPLA497‐L498ins4* and *MPLW515RQ516E*. Each group of cells was cultured without IL‐3 for 48 h, then total proteins were extracted. The total protein and phosphorylation protein of JAK2, STAT1, STAT3, STAT5, AKT, and ERK1/2 were examined by western blotting with the respective antiphospholipid‐specific antibodies. β‐actin was used as housekeeping protein for each group. Western blot analysis was repeated twice by Simple Western. Original figures of WB were given in Figure S2

To determine the activation of signaling pathways in *MPLA497‐L498ins4* and *MPLW515RQ516E* mutants, we further detected the phosphorylated MPL‐downstream signal proteins on Ba/F3 cells, including JAK2/STAT, PI3K/AKT, and MAPK/RAS pathway. Each group of cells was starved without IL‐3 for 48 hours. After 48 hours, total protein was extracted. We observed the increase of phosphorylated proteins in two atypical MPL mutants’ cells compared to *MPLWT* (Figure 5). The phosphorylated proteins referred to JAK2/STAT increased obviously in mutations of *MPL* (*A497‐L498ins4*, *W515RQ516E*, and *W515L*). In addition, the phosphorylated AKT and p44/42 MAPK involved in PI3K/AKT and MAPK/RAS pathway were also increased in two novel *MPL* mutations. *MPLWT*, empty vector, and un‐transfected Ba/F3 cells were used as negative control which activated proteins rarely without cytokine stimulation. The relative amount of each protein in each group is shown in Table S2.

Altogether, these results showed that two atypical *MPL* mutants can lead to cytokine‐independent cell growth, increase of TPO sensitivity, and G1/S‐phase transition on Ba/F3 cells via activating the JAK2/STAT and its downstream pathway. *MPLA497‐L498ins4* and *MPLW515RQ516E* mutations are gain‐of‐function mutations and closely associated with the pathogenesis of TN‐MPNs.

## 4 DISCUSSION

Most of the atypical *MPL* mutations described in many articles are missense mutations; however, in this study, we found two unusual mutated types of *MPL* exon10, an insertion mutation *MPLA497‐L498ins4*, and a double‐point mutation *W515RQ516E*. The *MPLA497‐L498ins4* was homozygous status in these two ET patients without disease progression which differs from the fact that *MPLW515K/L* mutation is usually heterozygous in the early period, but can become homozygous status during the disease progression.^18^ In our study, the *MPLA497‐L498ins4* and *W515RQ516E* are gain‐of‐function mutations inducing cytokine‐independent growth advantages in vitro and may be other disease‐causing factors for triple‐negative MPN. Many atypical MPL mutations have been verified to be activating mutations and may have oncogenic potentials, including *S204P*, *Y591D*, *W515R*, *W515A*, and so on. However, it is worthy to note that not all atypical mutations have biological functions. Previous research also demonstrated that *MPLA519T*, *L510P*, and *A506T* mutations were not gain‐of‐function mutations, and may be functioned as regulators or co‐occurred with other genetic events.^17^

Insertion or deletion mutations of MPL showed a low frequency of occurrence in MPN patients. Besides the *MPLA497‐L498ins4* in our study, only three insertion or deletion mutations have been reported, including the *HLdelinsVISLVT*, *W515‐P518delinsKT*, *T496‐A497ALVIins*, and* V502delinsS* (a germline mutation).^19^, ^20^, ^21^, ^22^ The insertion mutations of *MPLT496‐A497ALVIins* and *MPLA497‐L498ins4* are the same mutation discovered by different research teams coding for the same final sequence of LVTALVIAHLVLGLSAVLGLLLLRWQFP. The two different naming methods are mainly due to the fact that the mutation sequence contains the sequence (GCTCTG) which was overlapped with wild‐type sequences (GCTCTGCATCTAGTG). All three patients carrying *MPLA497‐L498ins4* mutation are presented as homozygous mutations. The insertion and deletion mutation *MPL HLdelinsVISLVT* is a gain‐of‐function mutation resulted in a strong constitutive activation of STAT5 in γ‐2A cells. It was also found that the activation of STAT5 was not inhibited after replacing Leu 515 with Trp in *HLdelinsVISLVT* mutant. Structural stability of transmembrane domain is critical for TPO‐R to perform its normal function. The *L498* transmembrane domain (TM) positions can maintain the activation of TM dimer interface and the *H499* is protective against the constitutive activation driven by several other mutations.^23^, ^24^, ^25^ We inferred that the *MPLA497‐L498ins4* mutant, inserted four amino acids from A497 to L498 amino acid, may change the normal structure of TPO‐R by destabilizing the stability of the TM dimer interface of TPO‐R, leading to an automatic dimerization in the absence of TPO. Further structural analysis will be required to identify the actual structure of *MPL* insertion or deletion mutations.

The *MPLW515RQ516E* mutation has never been reported in ET patients. The *W515R* single‐site mutation has been well studied to be associated with the pathology of MPN,^14^ but the *W515R* combined with *Q516E* has never been discussed. We explored the function of double‐point mutation *W515RQ516E* in vitro, and the result showed that it was sufficient to promote cell growth in the absence of cytokine. It is interesting that the biological function of *MPLW515RQ516E* is totally different from *W515LQ516W* or *W515KQ516W* which is ineffective to trigger the autonomous proliferation in vitro.^26^ Structural study demonstrated that the second‐site mutation *Q516W* can reverse TPO‐R dimerization effect and tilt angle changes induced by *W515K/L* mutations, indicating that the amino acid *Q516* has the potential to mediate helix interactions.^27^ We wondered whether the *Q516E* mutation was involved in the auto‐proliferation effect of *MPLW515RQ516E*. Then, we constructed the single‐site mutation *Q516E* and identified its function. Results showed that mutation of *Q516E* alone was a weak activating mutation and sufficient to promote proliferation on Ba/F3 cells in the absence of IL‐3 but weaker than classical *W515L* and other two atypical *MPL* mutations in our study. This outcome was consistent with the effect of *MPLW515RQ516E* mutation. However, we did not observe a duplicate effect on cell proliferation of W515RQ516E double‐point mutation. We inferred that the *Q516E* may play a role in maintaining or modulating the steady state of TPO‐R caused by *W515R* mutation rather than augmenting the proliferation effect of *W515R*. Considering two polarized consequences between *MPLW515RQ516E* and *W515LQ516W,* we analyzed the properties of two mutations and we found that they differ greatly in the nature of amino acids. Under normal condition, the site 516 of the wild‐type protein is Glutamine (Q), a hydrophilic neutral amino acid. In our study, it was substituted by a hydrophilic acidic amino acid, glutamic acid (E). But in J‐P Defour's study, the Glutamine was replaced by tryptophan (W), a hydrophobic neutral amino acid. The hydrophobic neutral amino acid, tryptophan (W) at TM domain is critical to modulate TPO‐R dimerization and activation while the negatively charged hydrophilic amino acid of Glu may be more effective in maintaining the dimerization of the TM α‐helix caused by *W515K/L/R* rather than the reversion effect. Many questions about TPO‐R structure remain unresolved, thus further biophysical studies will be required to identify the interaction of different amino acids at the site of 515 and 516.

In our study, the percentage of cells entering to S phase was increased in atypical *MPL* mutations in the absence of IL‐3 or TPO, indicating that *MPLA497‐L498ins4* and *MPLW515RQ516E* mutants promoted G1/S transition on Ba/F3 cells. Previous works have demonstrated that promotion of G1/S transition induced by *MPLW515L/K* mutation was related to the increased expression of cycle‐regulator proteins including E2F1, cyclins D1 and D3, PCNA, cyclins A and E.^17^ Even though we did not detect the expression of cycle‐regulator protein in our study, we found that JAK2/STAT and its downstream signaling pathways were strongly activated in *MPLA497‐L498ins4* and *W515RQ516E* mutants after cytokine withdrawal. These results suggested that these activated kinases caused by two atypical *MPL* mutations were similar to the effects induced by mutation of *JAK2 V617F* or *MPLW515K/L*. The spontaneous activation of signal pathway transduction in JAK2/STAT, PI3K/AKT, and MAPK/RAS was involved in the cell autonomous growth and cell cycle transition of two novel MPL mutations.

Altogether, our study showed that the *MPLA497‐L498ins4* and *W515RQ516E* were causative mutations in triple‐negative ET cases. With the development of NGS, more and more atypical mutations have been reported. The mutation sites of *MPL* gene are in great diversity and the function of them also varies considerably. Thus, we cannot confirm the newly discovered *MPL* mutation as a driver mutation until further investigations are performed and the mechanisms underlying these mutations are demonstrated.

## CONFLICT OF INTEREST

The authors declare no conflict of interest.

## AUTHOR CONTRIBUTIONS

H. W. and X. C. conceived the study. J. X., X. C., and F. G. performed most of experiments and statistical analysis. J. X. and X. C. wrote the manuscript. R. H., T. T., Y. Z., L. F., J. H., G. Z., and W. Y. performed clinical analysis and some experiments.
