# Supplementary material for: Two activating mutations of MPL in triple‐negative myeloproliferative neoplasms
Source: Cancer Med. 2019 Jul 11;8(11):5254–63. doi: 10.1002/cam4.2387 (PMC6718619; doi:10.1002/cam4.2387)
Supplement: Supplementary file 1 [file CAM4-8-5254-s001.zip › cam42387-sup-0001-Supinfo.docx]

This file contains Supplementary Tables 1-2 and Supplementary Figure1-2. Supplementary Table 1 listed the detail source of major regents and kits used in our study. Supplementary table 2 contains the relative amount of each protein in different groups, we determined it by the Compass for SW software.

### Supplementary figure 1: FACS gating strategy for cell cycle analysis of Ba/F3 cells under various concentration of TPO. Representative plot showing gating strategy on Ba/F3 cells. Cells characterized with a high FSC/SSC profile were selected and cells presenting a lower FSC/SSC profile were regarded as debris or noise.(supplementary figure 1-A) . Cells tagged with PI should be distributed in a linear correlation with AUX signal. Nonlinear related cells were adherent cells or cell fragments and then removed.(supplementary figure 1-B). The percentage of G1, G2 and S phase was determined by the Modfit software. The results were derived from 3 biologically repeated experiments. Supplementary figure 1-C to 1-E shows the histogram for cell cycle analysis under 0ng/ml,0.1ng/ml and10ng/ml TPO, respectively. (related to Fig 4). Supplementary figure 2 from2- 1 to 2-13 contains the uncropped images of western blot data of STAT1, p-STAT1, STAT3, p-STAT3, STAT5, p-STAT5, AKT, p-AKT, ERK, p-ERK, JAK2, p-JAK2 and β-actin，respectively.

|  | **origin** | **catalog numbers** |
| --- | --- | --- |
| **pCDH-MCS-T2A-copGFP-MSCV** | SBI, USA | CD523A-1 |
| **pMD^TM^19-T vector Cloning Kit** | Takara, Japan | 6013 |
| **Anti-JAK2** | CST, USA | 3230S |
| **Anti -STAT1** | CST, USA | 9172S |
| **Anti -STAT5** | CST, USA | 94205S |
| **Anti -STAT3** | CST, USA | 12640S |
| **Anti -AKT** | CST, USA | 4691S |
| **Anti -ERK** | CST, USA | 4695S |
| **Anti -p-STAT1(Tyr701）** | CST, USA | 9167S |
| **Anti -p-STAT3(Tyr705)** | CST, USA | 9145S |
| **Anti -p-STAT5(Tyr694)** | CST, USA | 9359S |
| **Anti -p-p44/p42MAPK (Erk1/2) (Thr202/ Tyr204)** | CST, USA | 4370S |
| **Anti -p-JAK2(Tyr1007/1008)** | Abcam, USA | AB32101 |
| **Anti -Beta-Actin** | Abcam, USA | AB8227 |
| **Anti -p-AKT(Thr308)** | Arigo, China | SRG51559 |
| **PE-Mouse anti-Human CD110** | BD pharmingen^TM^ ,USA | 562159 |
| **RPMI 1640** | Gibco, USA | C11875500BT |
| **DMEM** | Gibco, USA | C11995500BT |
| **DPBS** | Gibco, USA | C14190500BT |
| **Fatal Bovine Serum, Certified** | Gibco, USA | 16000-044 |
| **Lipo D293^TM^Reagent** | SignaGen | SL100668 |
| **RIPA buffer** | Thermo Scientific, USA | 89901 |
| **Pierce™ Protease and Phosphatase Inhibitor Mini Tablets** | Thermo Scientific, USA | A32961 |
| **Cell Cycle and Apoptosis Analysis Kit** | Bey time, China | C1052 |
| **Wes separation 12-230kDa 8*25 Capillary Cartridges** | Protein Simple, USA | SM-W004-1 |
| **Standard Pack 1** | Protein Simple, USA | PS-ST01-8 |
| **Anti-Rabbit Detection Module** | Protein Simple, USA | DM-001 |
| **EndoFree Maxi Plasmid Kit** | TIANGEN, China | DP108 |
| **Cell counting Kit-8** | DOJINDO, Japan | CK04 |
| **Site-directed Mutagenesis kit** | Agilent Technologies, USA | 210518 |
| **Ba/F3 cell line** | Beijing, China | 3111C0001CCC000095 |
| **293 T cell line** | ATCC, USA | CRL-3216 |

**Supplementary table1: Details about the vectors, plasmid, buffer and kits.**

**Supplementary table2: The relative amount of proteins detected by Simple Western**

|  | **MPLA497-L498ins4** | **MPLW515RQ516E** | **MPLW515L** | **MPLWT** | **Empty vector** | **Ba/F3** |
| --- | --- | --- | --- | --- | --- | --- |
| **Beta-actin** | 14194 | 25285 | 19808 | 21908 | 19031 | 14734 |
| **JAK2** | 118542 | 141783 | 152861 | 111383 | 145615 | 257780 |
| **P-JAK2** | 40405 | 52007 | 65758 | 6272 | 179228 | 7065 |
| **STAT1** | 6777443 | 3552423 | 4148778 | 5717045 | 5199248 | 3894141 |
| **P-STAT1** | 96223 | 46974 | 89752 | 0 | 0 | 0 |
| **STAT3** | 229419 | 218815 | 227458 | 175239 | 115931 | 158163 |
| **P-STAT3** | 90067 | 93268 | 162211 | 12288 | 0 | 0 |
| **STAT5** | 2259640 | 3110625 | 3124480 | 1329679 | 2995849 | 3867042 |
| **P-STAT5** | 114624 | 95284 | 127302 | 23813 | 15619 | 31197 |
| **AKT** | 2576840 | 2757515 | 2947047 | 1890899 | 2379323 | 3021298 |
| **P-AKT** | 237733 | 236069 | 186604 | 32726 | 42819 | 0 |
| **ERK** | 4064041 | 3803366 | 3486977 | 1284711 | 2315017 | 2847608 |
| **P-ERK** | 859093 | 564277 | 508385 | 299999 | 115405 | 159279 |

**Supplementary Figure 1-A**

**
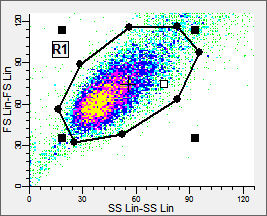
**

**Supplementary Figure 1- B**

**
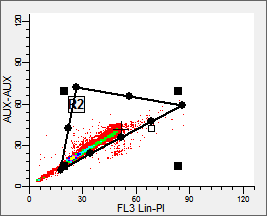
**

**Supplementary Figure1-C**

**
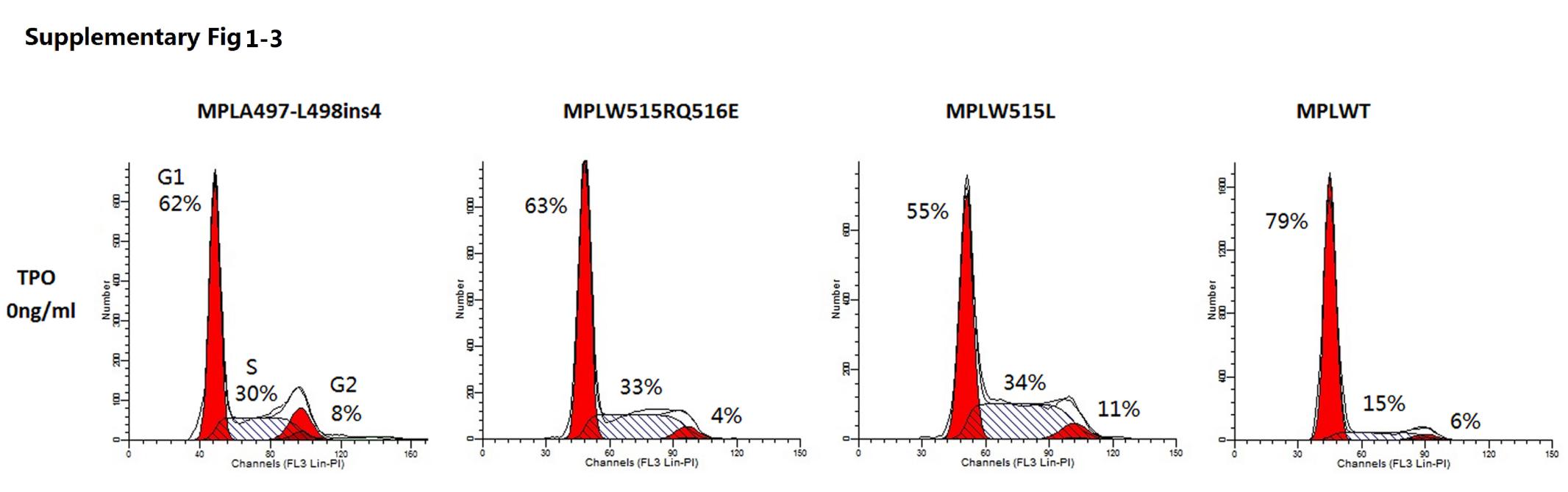
Supplementary Figure1-D**


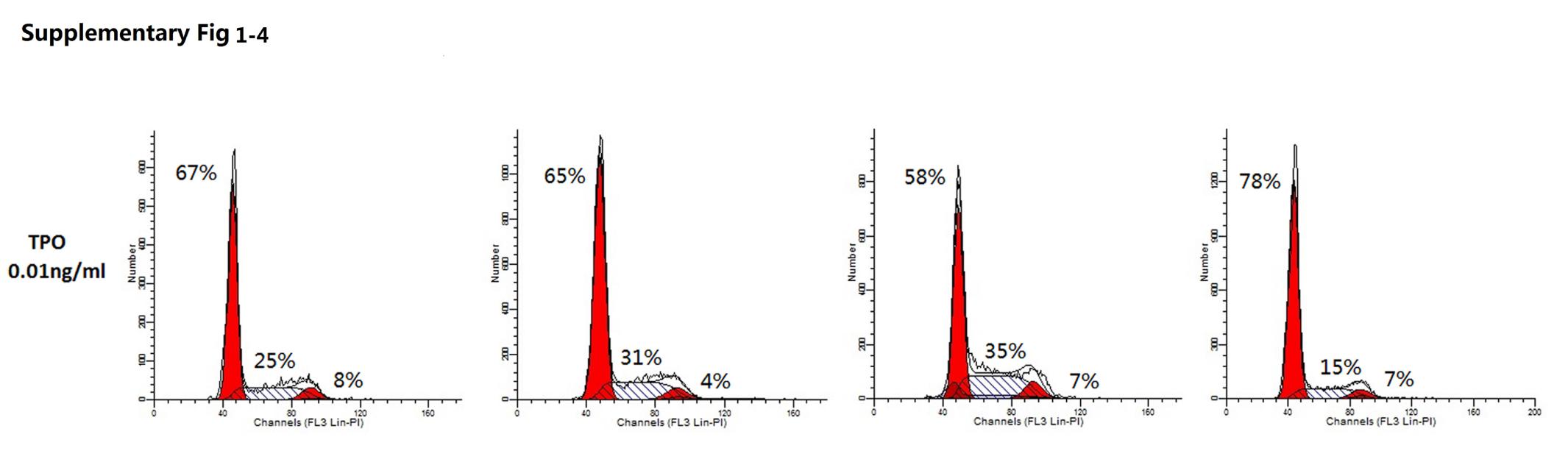


**Supplementary Figure 1-E**

**
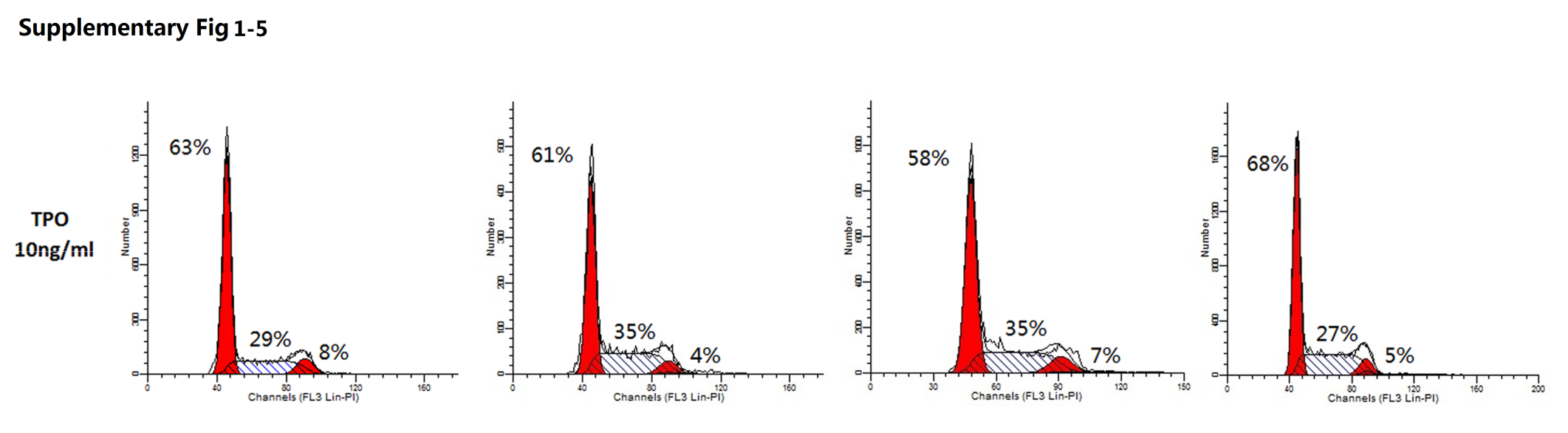
**

**Supplementary Figure 2- 1：uncropped WB images of STAT1**

**
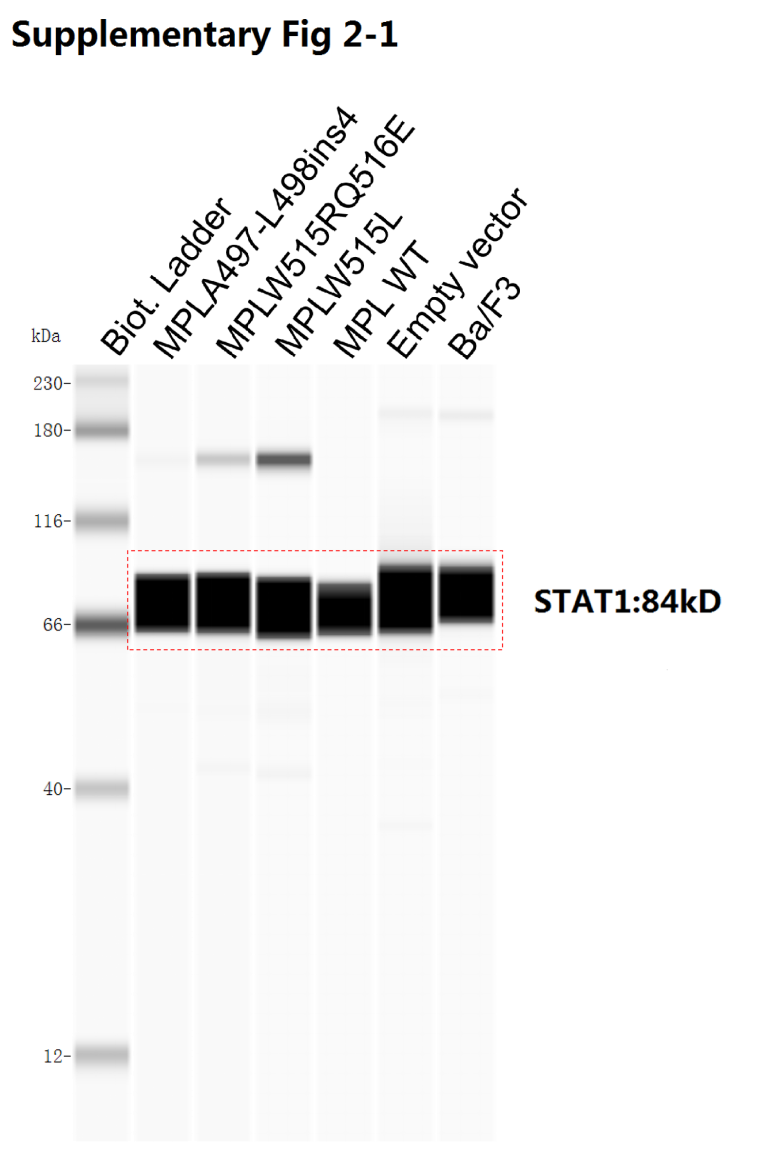
**

**Supplementary Figure 2- 2：uncropped WB images of p-STAT1**

**
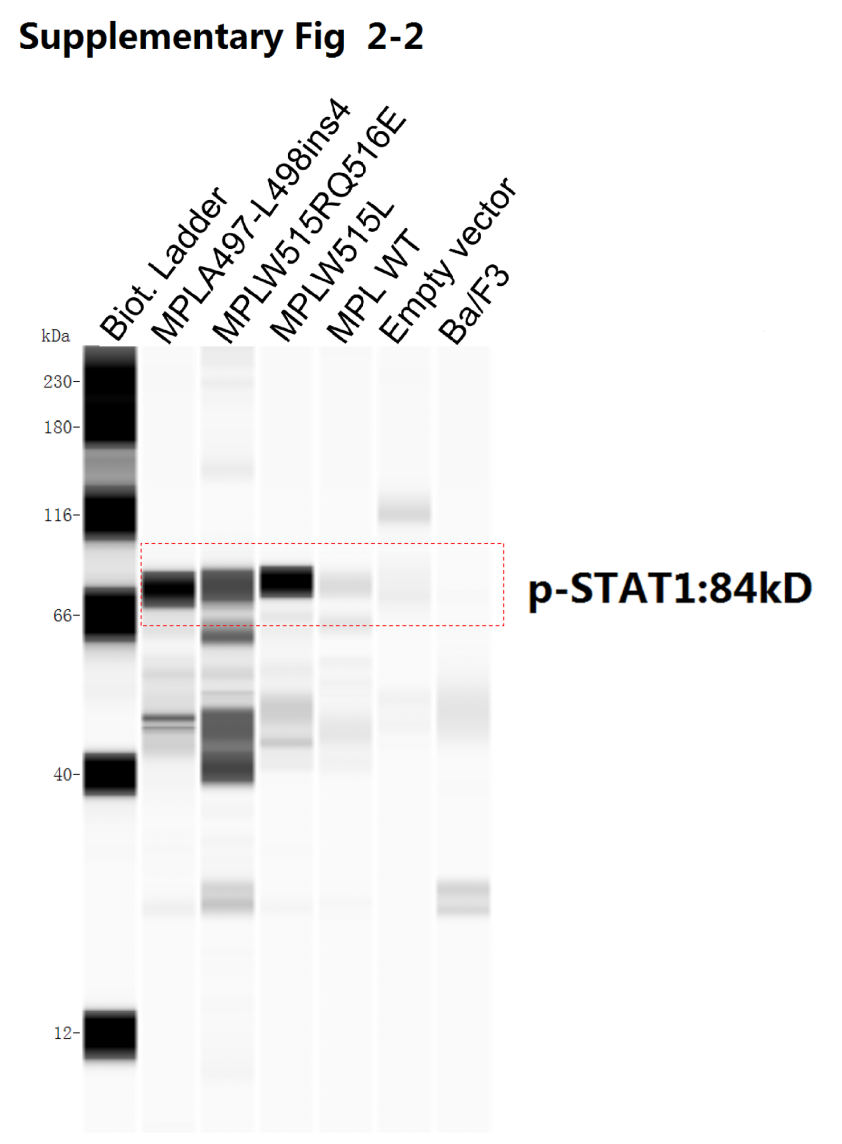
**

**Supplementary Figure 2- 3：uncropped WB images of STAT3**

**
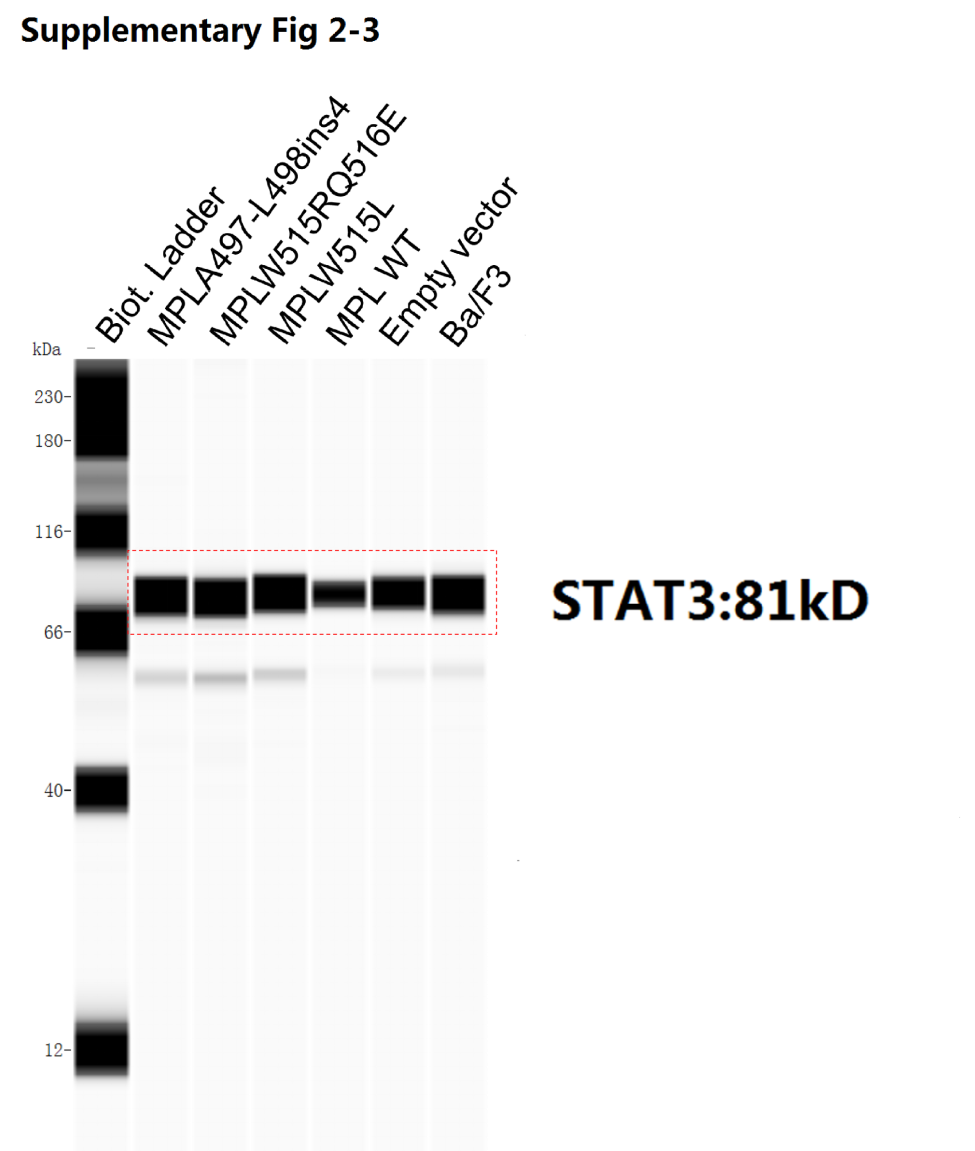
**

**Supplementary Figure 2- 4：uncropped WB images of p- STAT3**

**
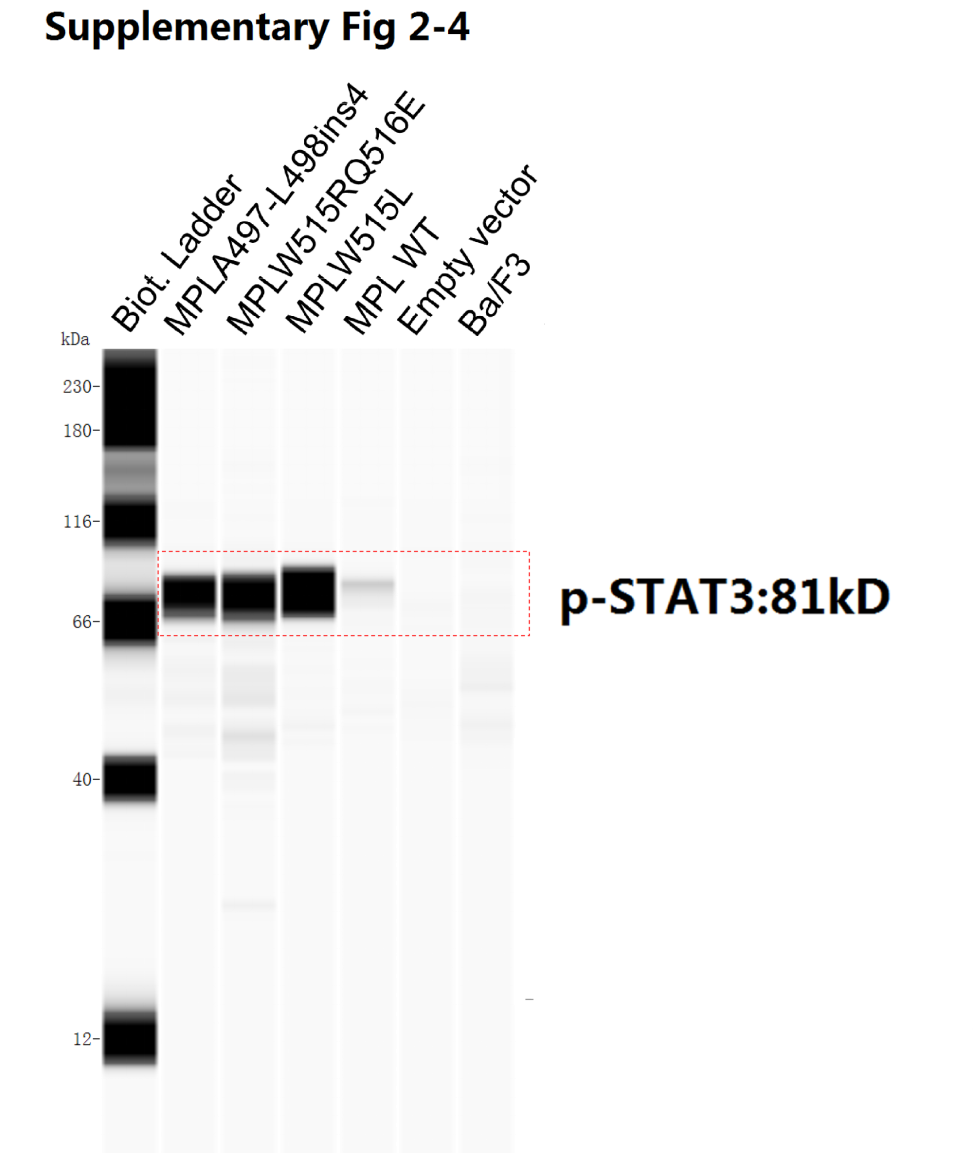
**

**Supplementary Figure 2- 5：uncropped WB images of STAT5**

**
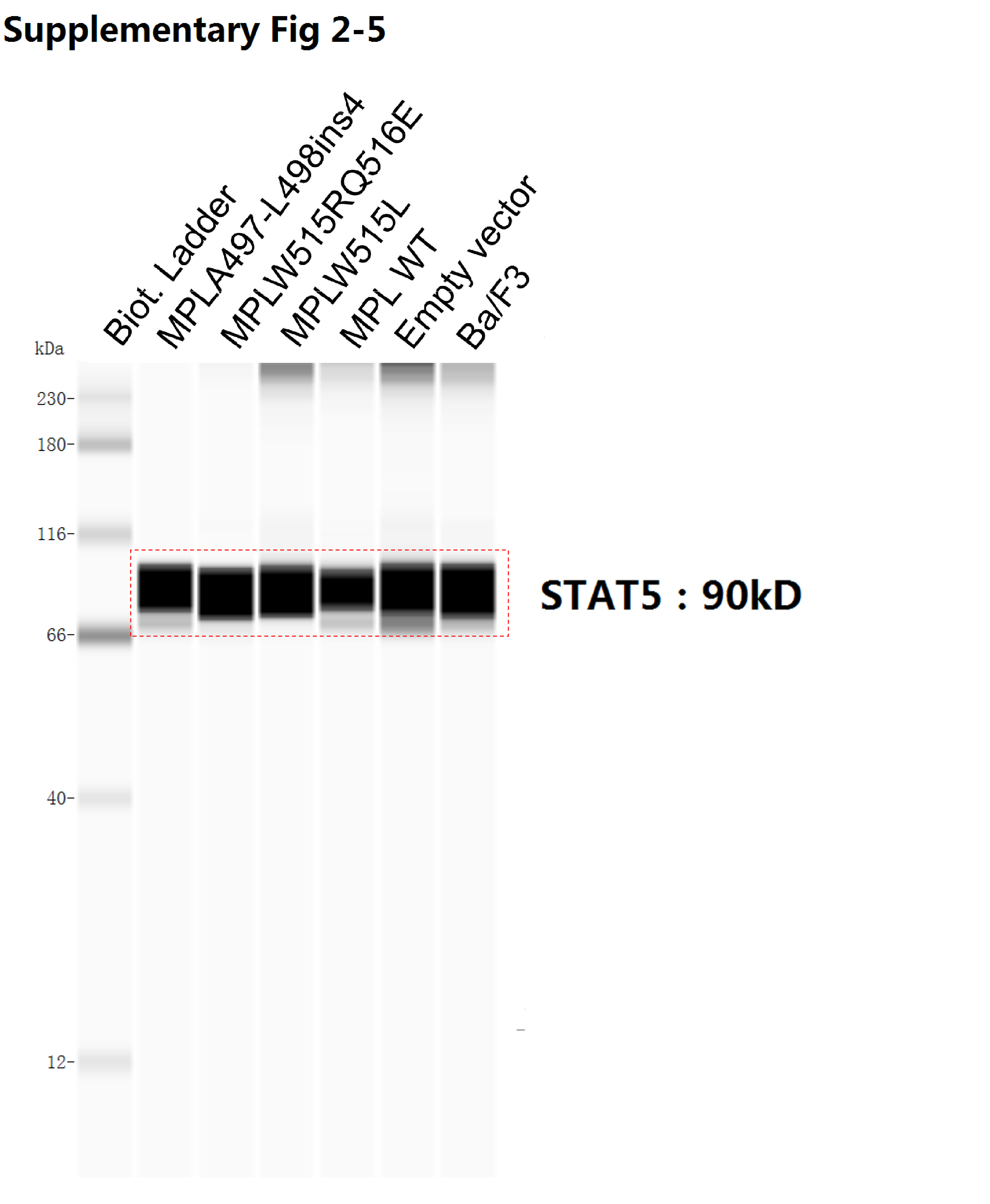
**

**Supplementary Figure 2- 6：uncropped WB images of p-STAT5**

**
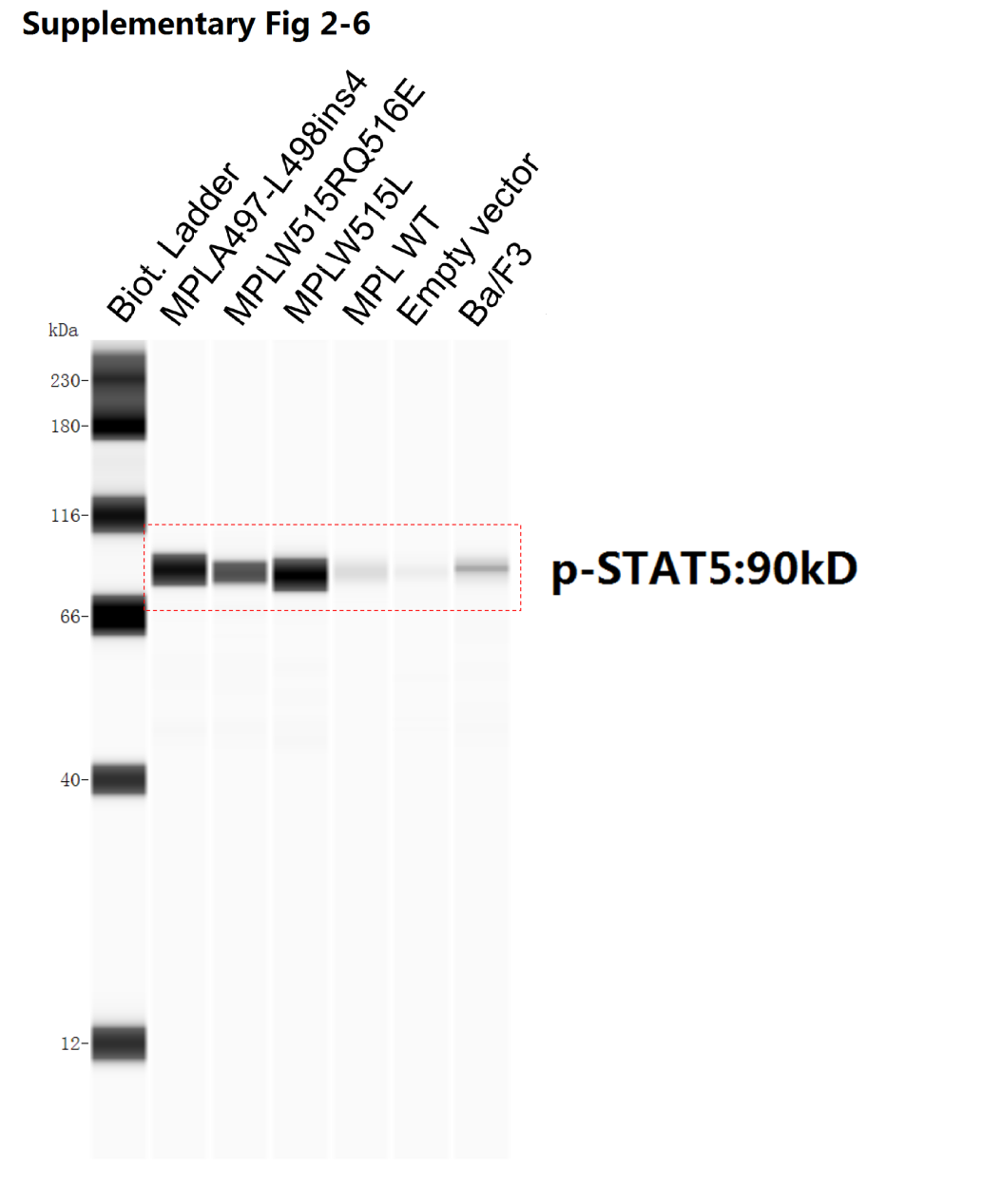
**

**Supplementary Figure 2- 7：uncropped WB images of ERK**

**
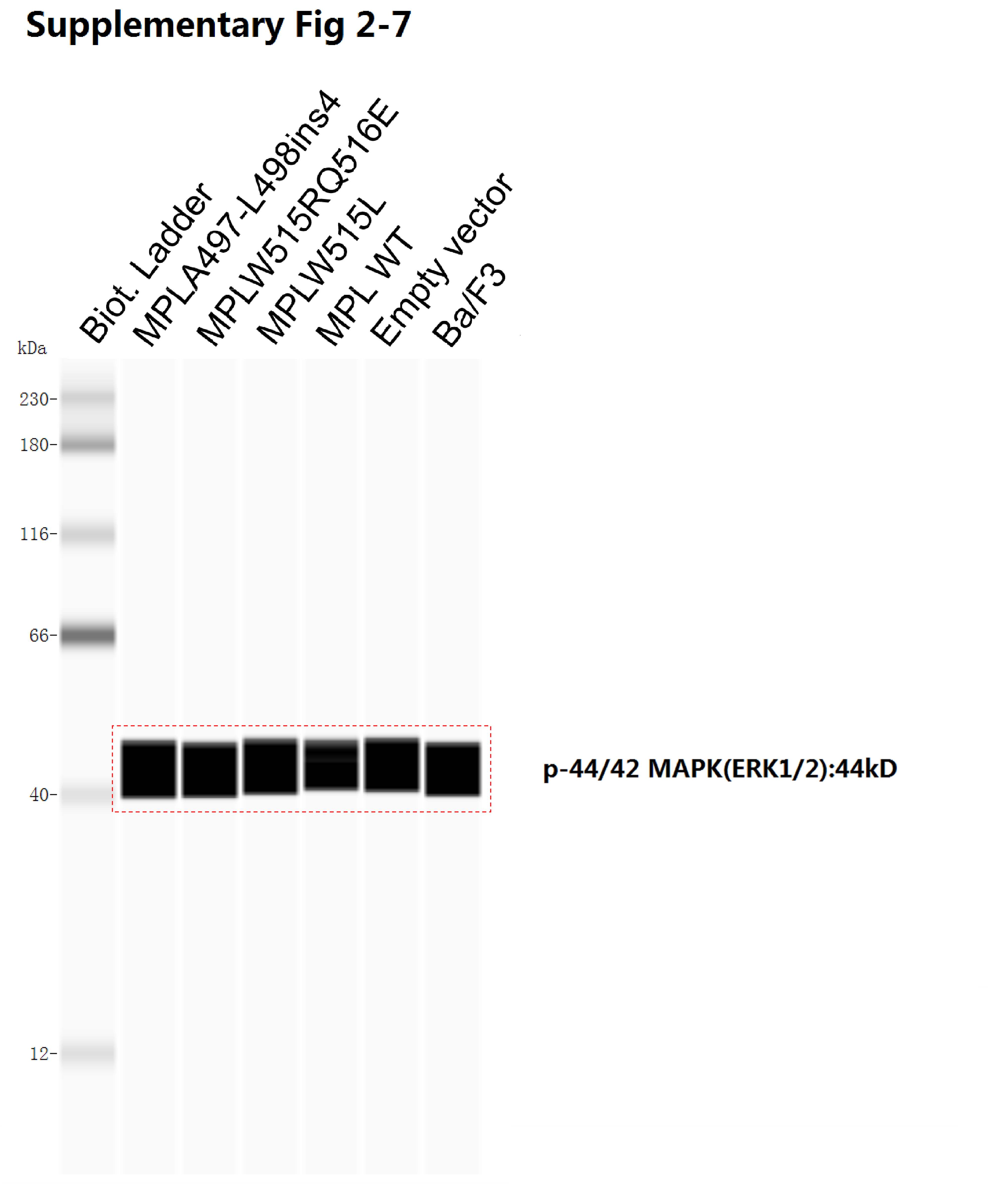
**

**Supplementary Figure 2- 8：uncropped WB images of p-ERK**

**
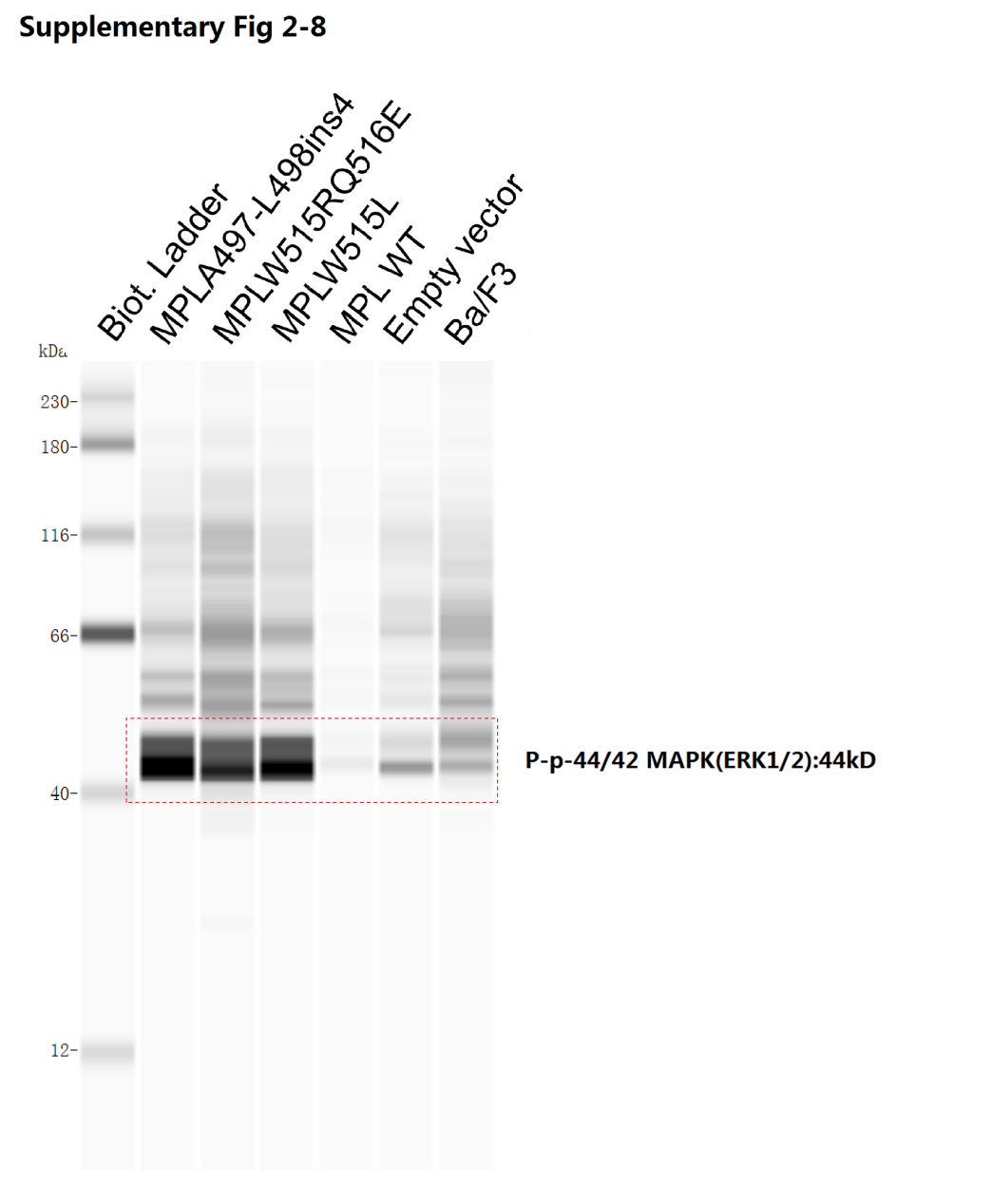
**

**Supplementary Figure 2- 9：uncropped WB images of AKT**

**
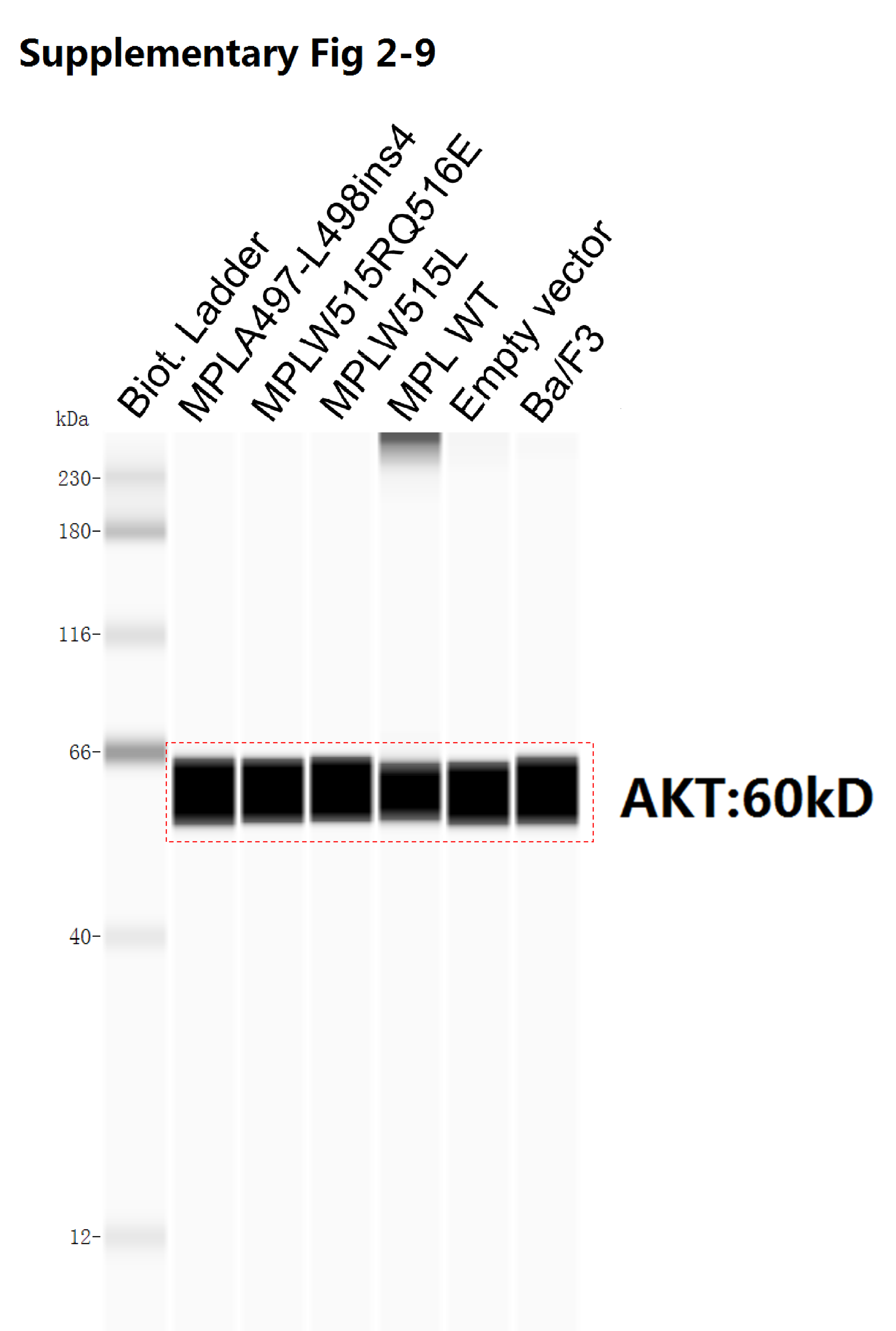
**

**Supplementary Figure 2- 10：uncropped WB images of p-AKT**

**
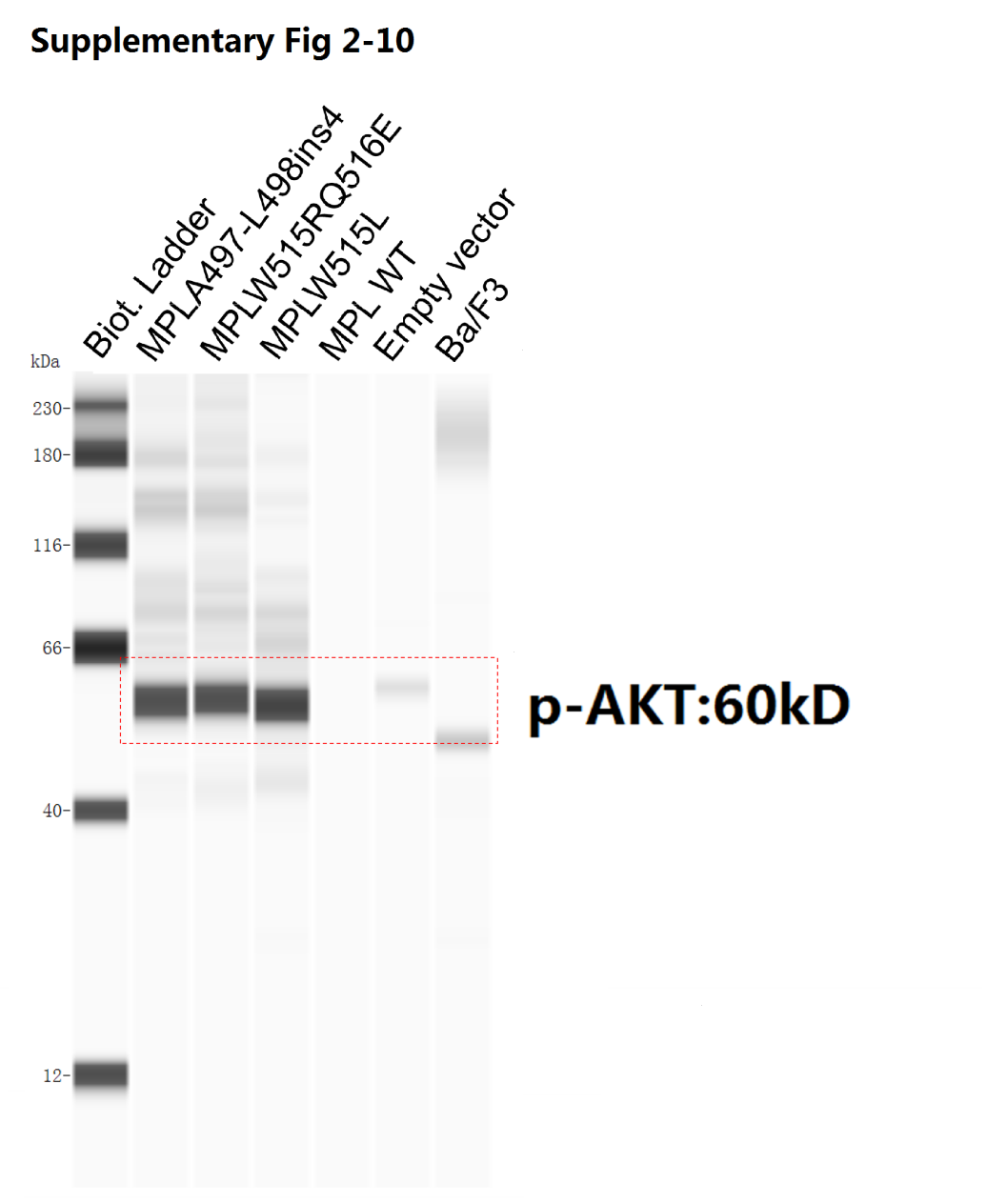
**

**Supplementary Figure 2- 11：uncropped WB images of JAK2**

**
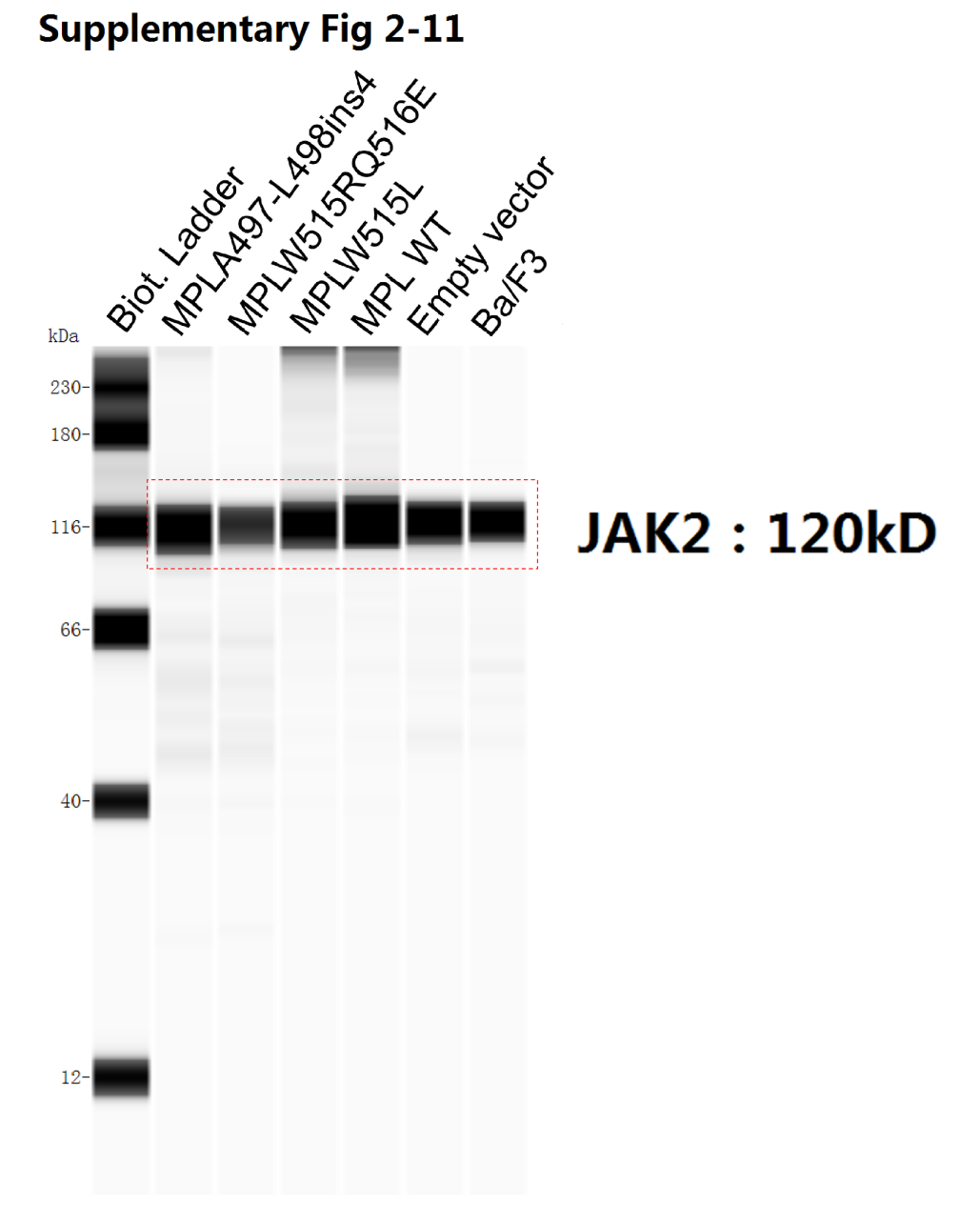
**

**Supplementary Figure 2- 12：uncropped WB images of p-JAK2**

**
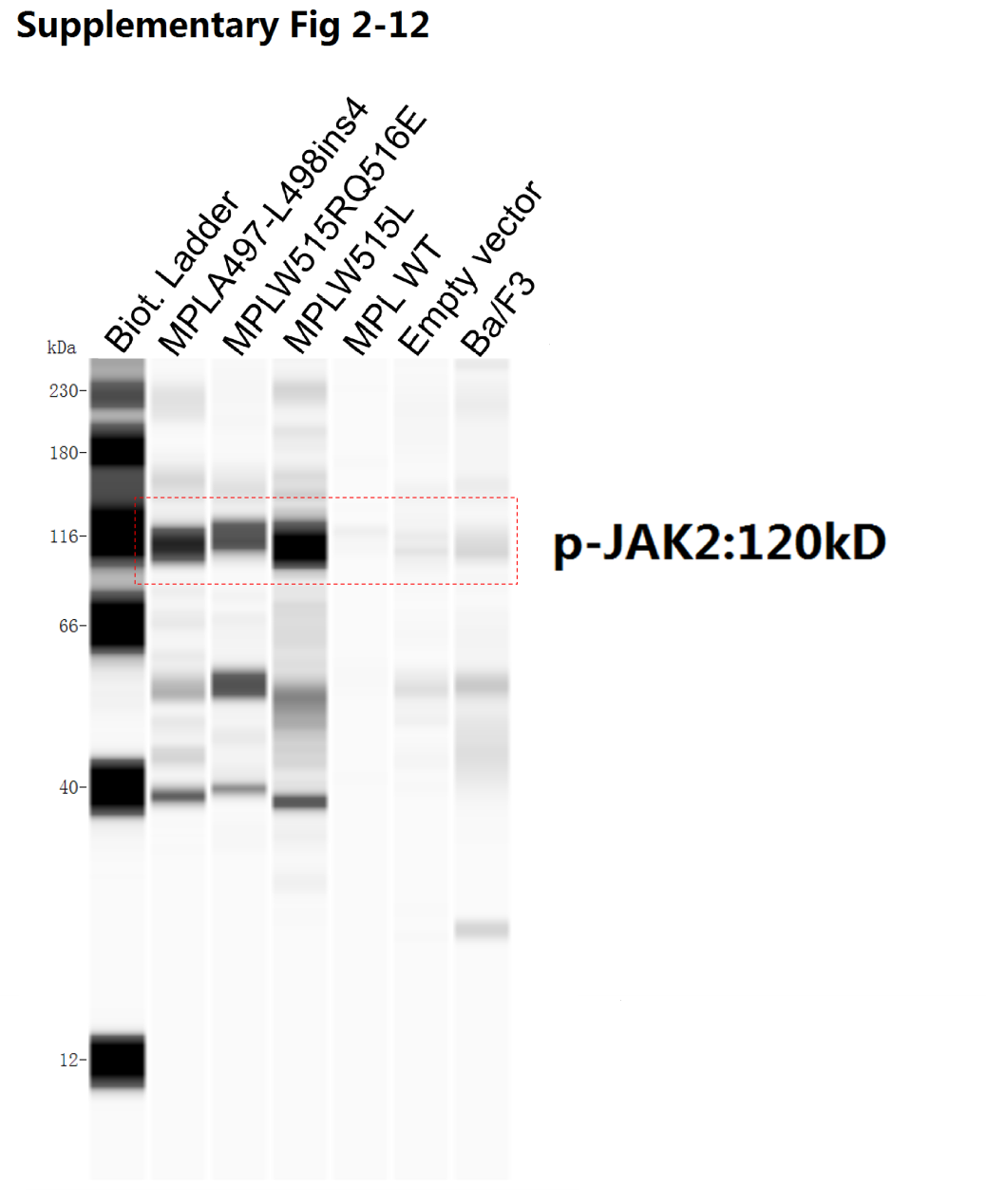
**

**Supplementary Figure 2- 13：uncropped WB images of β-actin**

**
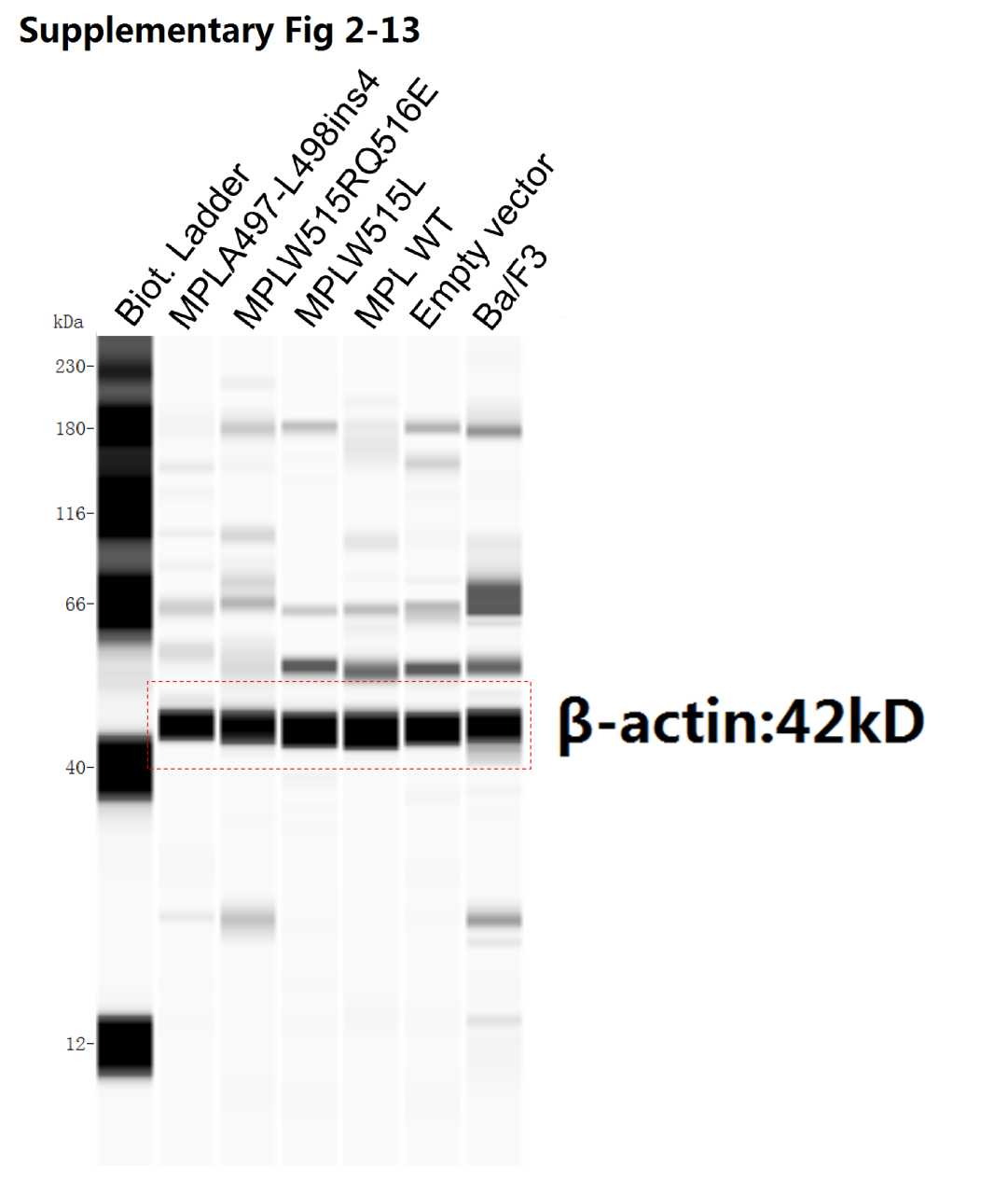
**
