# Supplementary figures and images for: Two activating mutations of MPL in triple‐negative myeloproliferative neoplasms
Source: Cancer Med. 2019 Jul 11;8(11):5254–63. doi: 10.1002/cam4.2387 (PMC6718619; doi:10.1002/cam4.2387)

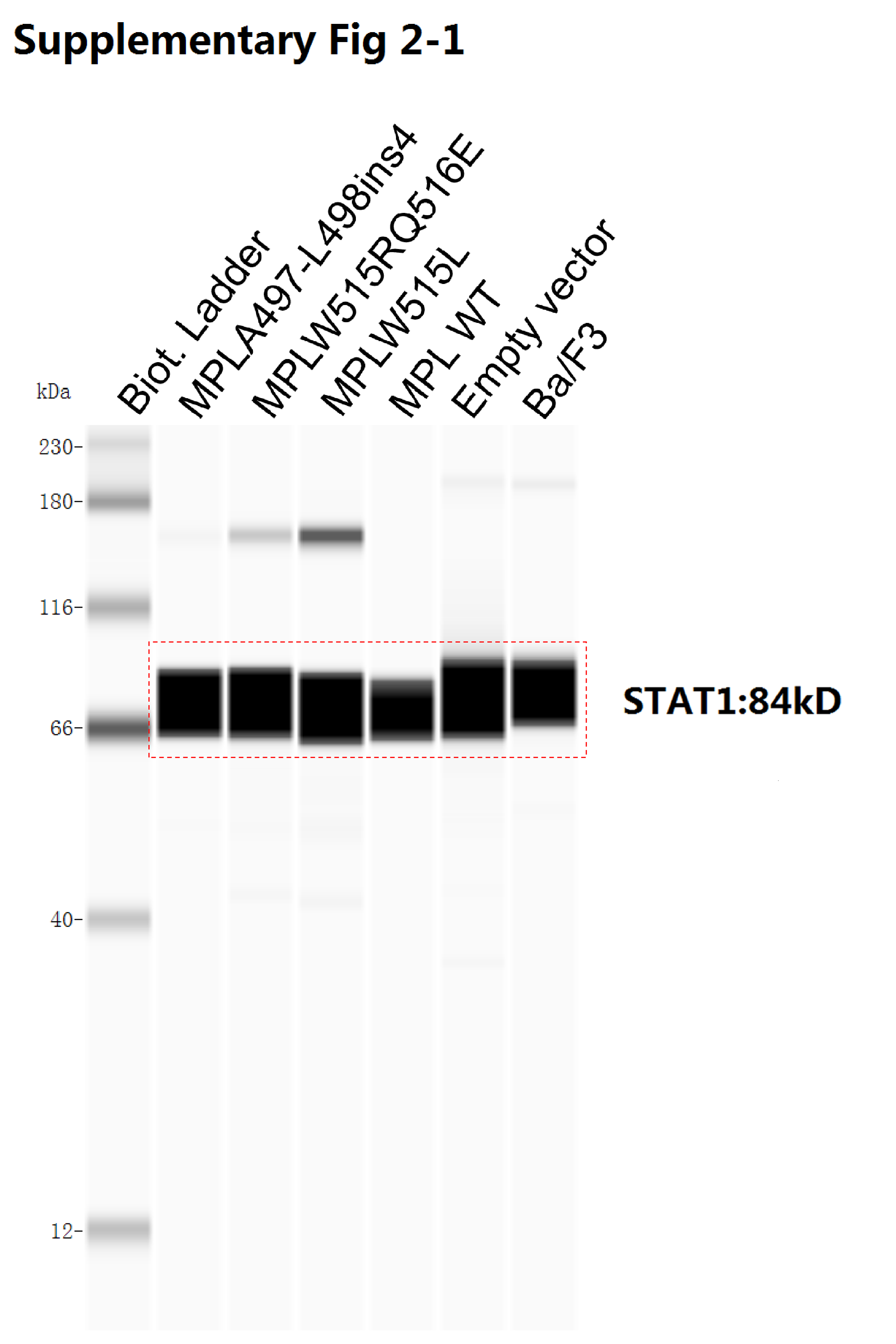

Supplement: Supplementary file 1 [file CAM4-8-5254-s001.zip › 1,stat1.tif]

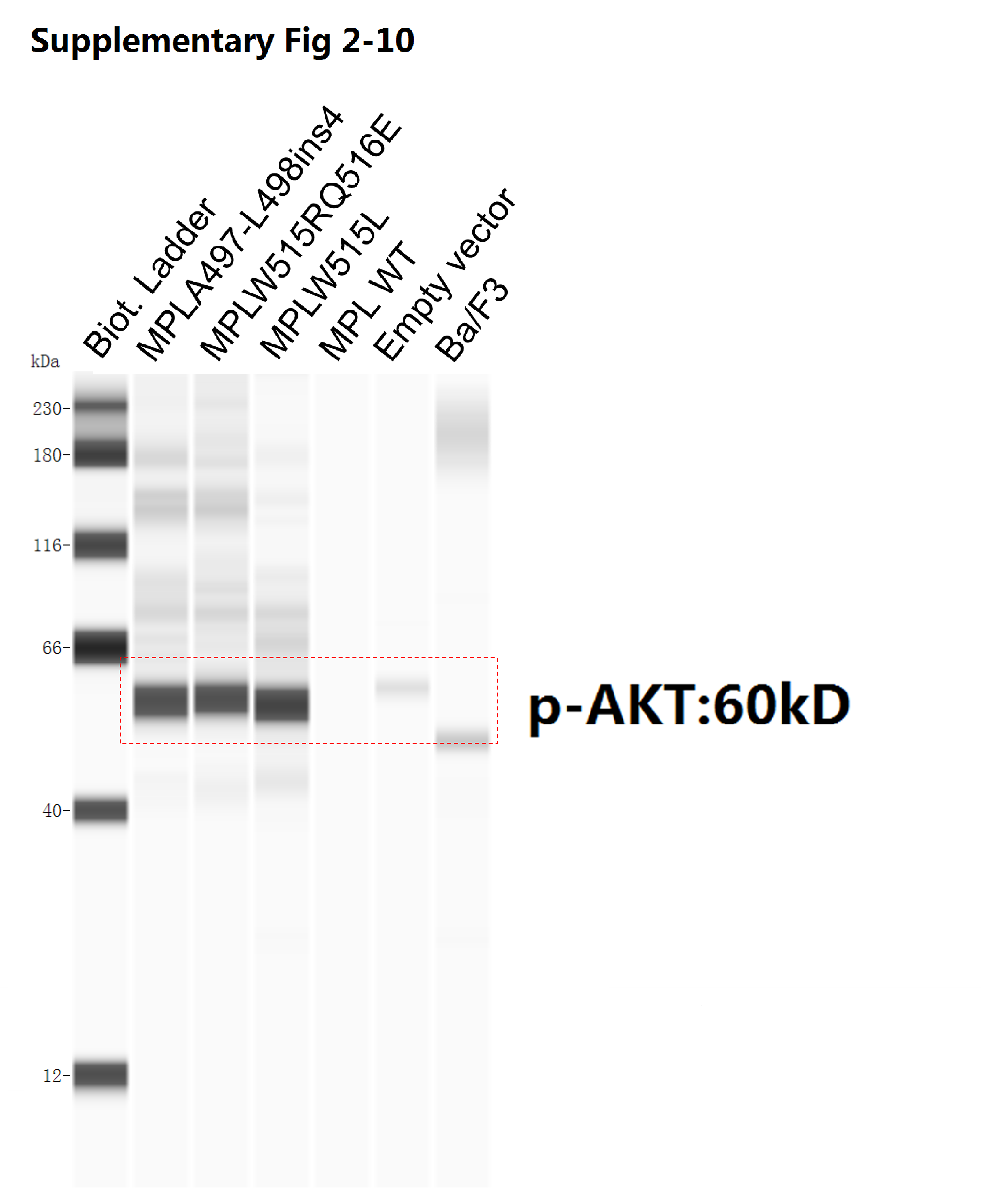

Supplement: Supplementary file 1 [file CAM4-8-5254-s001.zip › 10.P-AKT.tif]

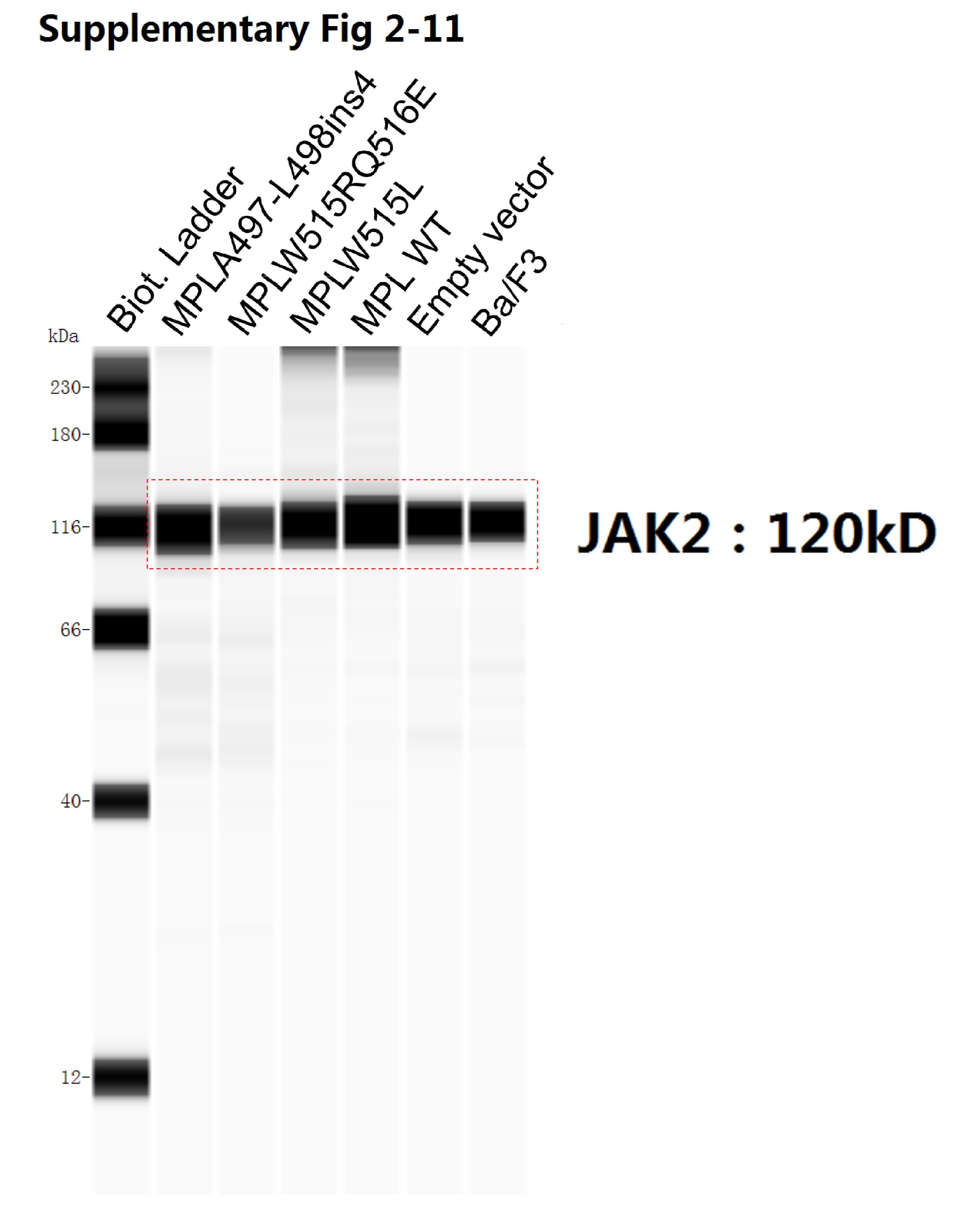

Supplement: Supplementary file 1 [file CAM4-8-5254-s001.zip › 11.JAK2.tif]

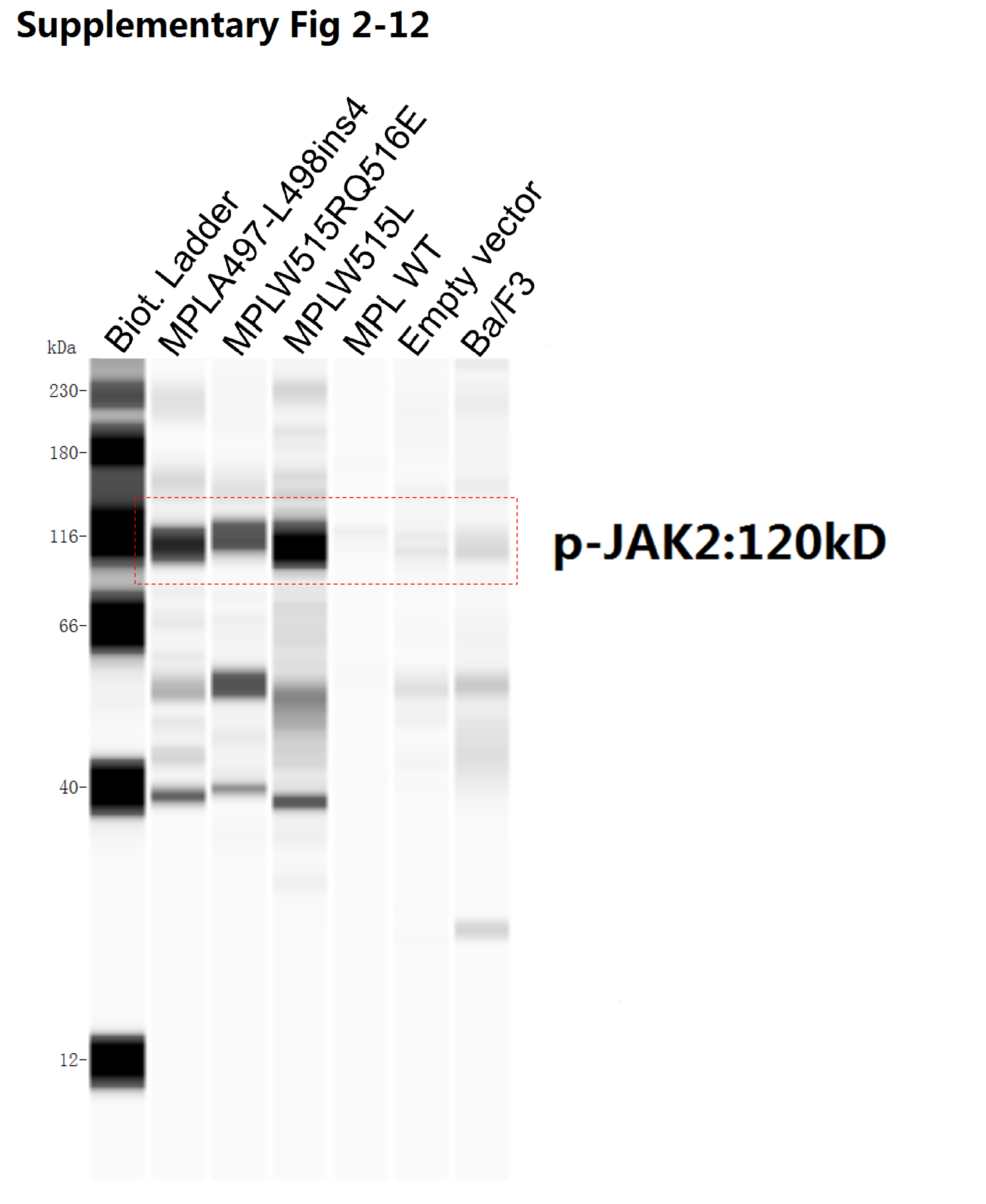

Supplement: Supplementary file 1 [file CAM4-8-5254-s001.zip › 12.p-JAK2.tif]

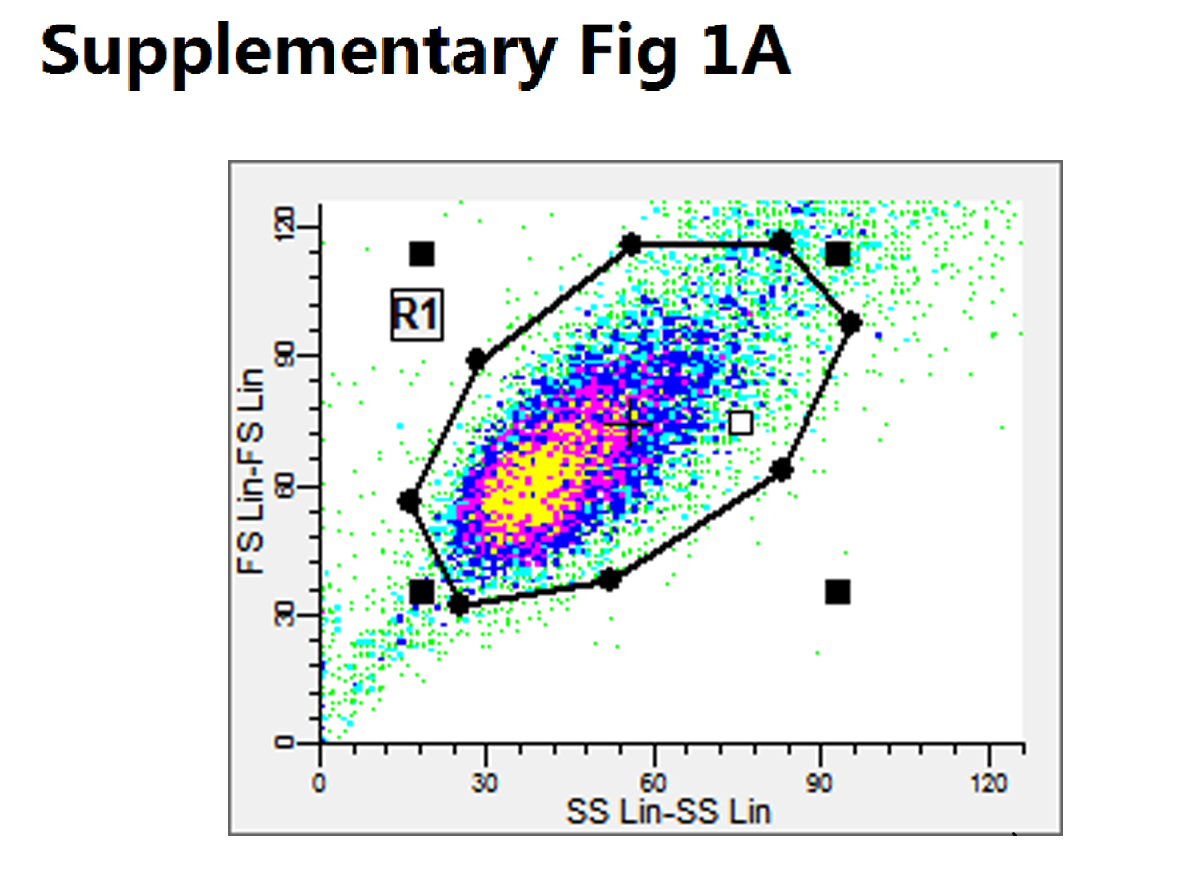

Supplement: Supplementary file 1 [file CAM4-8-5254-s001.zip › 1tif.tif]

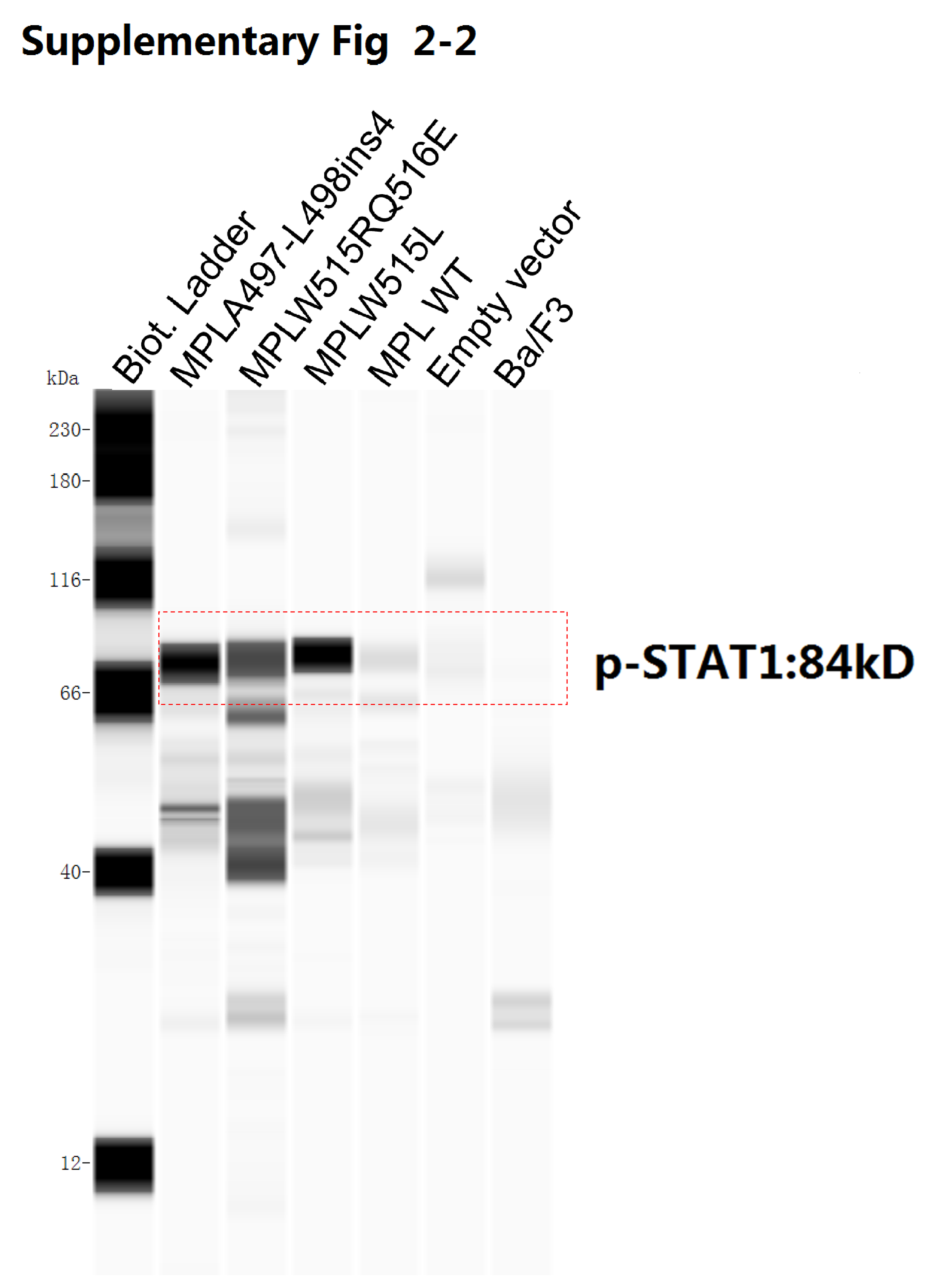

Supplement: Supplementary file 1 [file CAM4-8-5254-s001.zip › 2.p-stat1.tif]

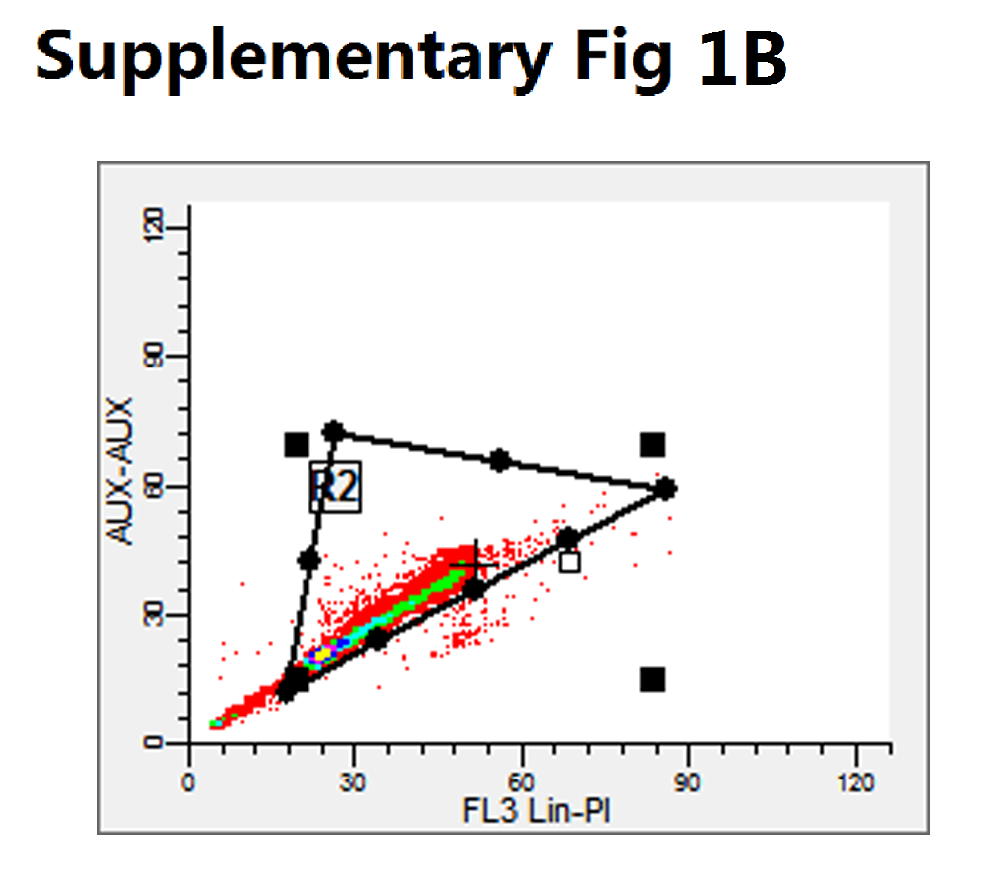

Supplement: Supplementary file 1 [file CAM4-8-5254-s001.zip › 2.tif]

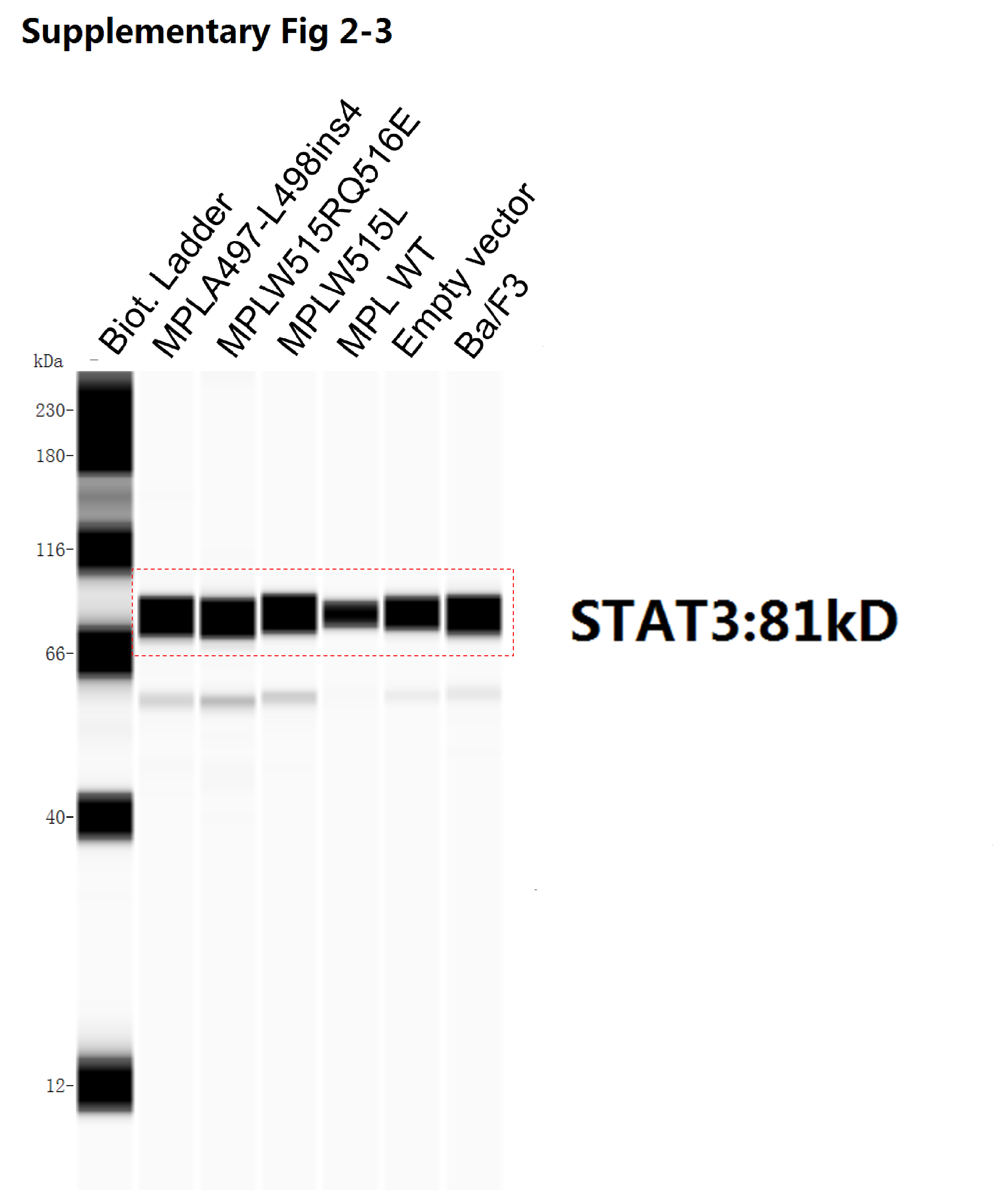

Supplement: Supplementary file 1 [file CAM4-8-5254-s001.zip › 3.stat3.tif]

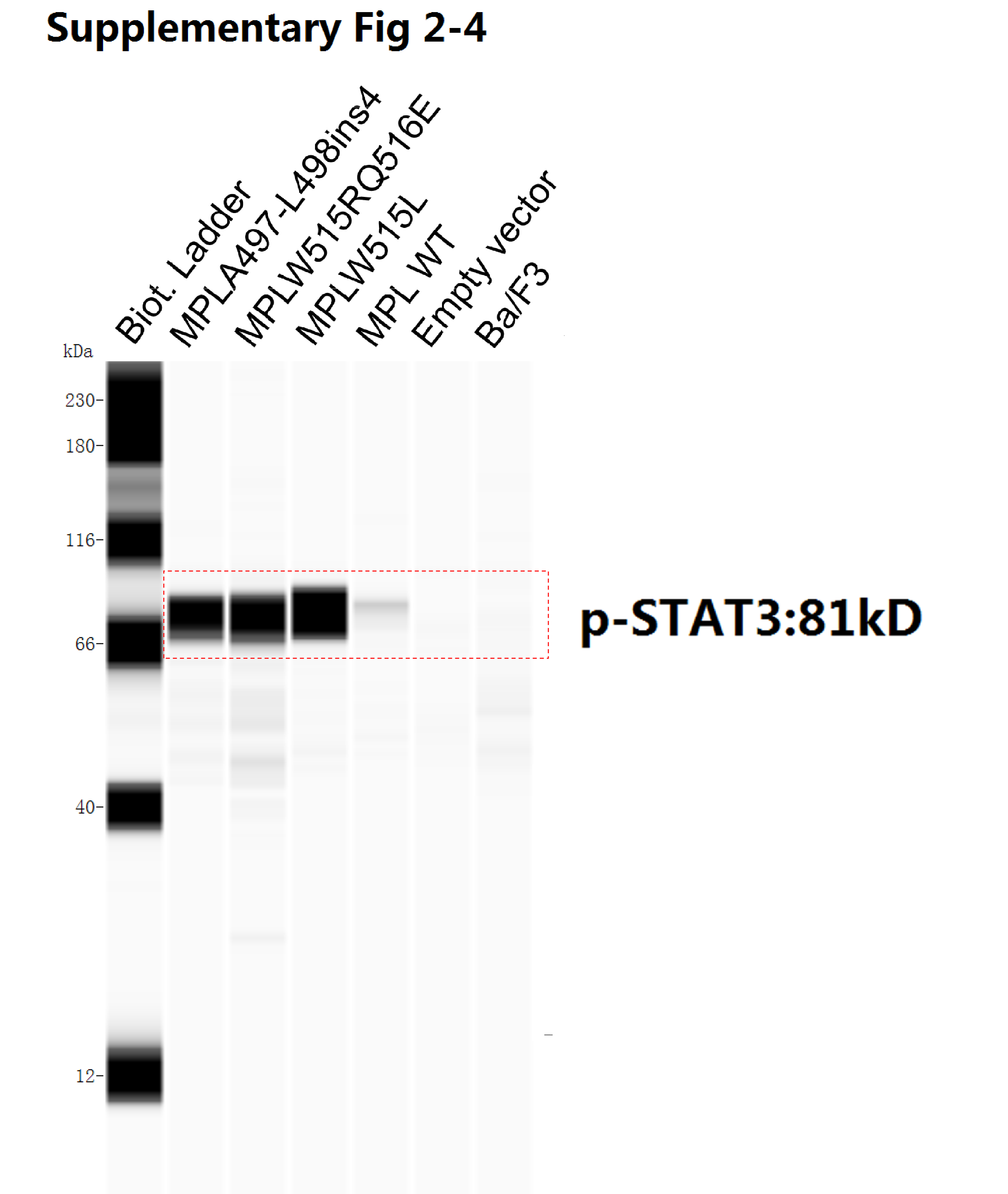

Supplement: Supplementary file 1 [file CAM4-8-5254-s001.zip › 4.p-stat3.tif]

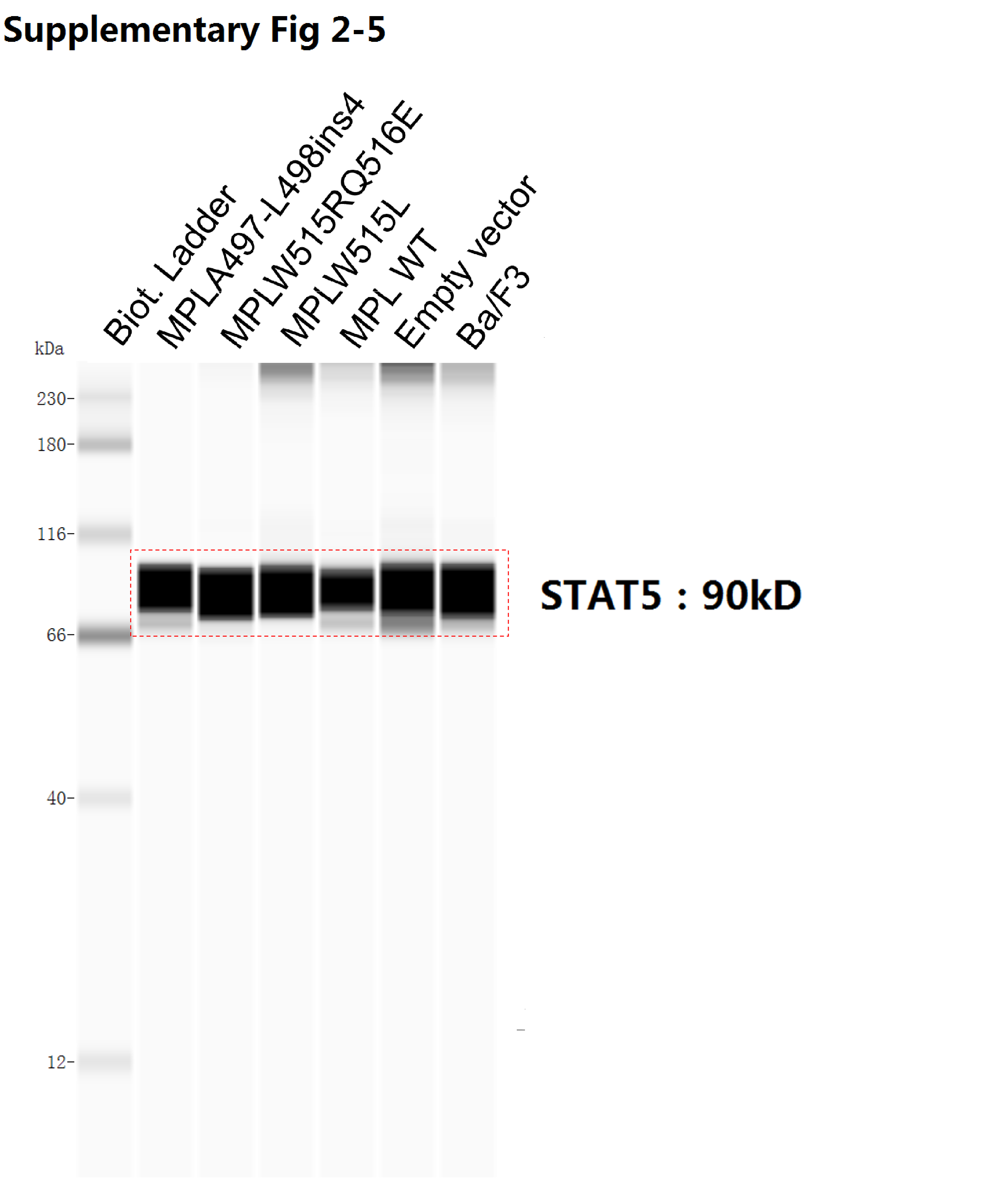

Supplement: Supplementary file 1 [file CAM4-8-5254-s001.zip › 5.stat5.tif]

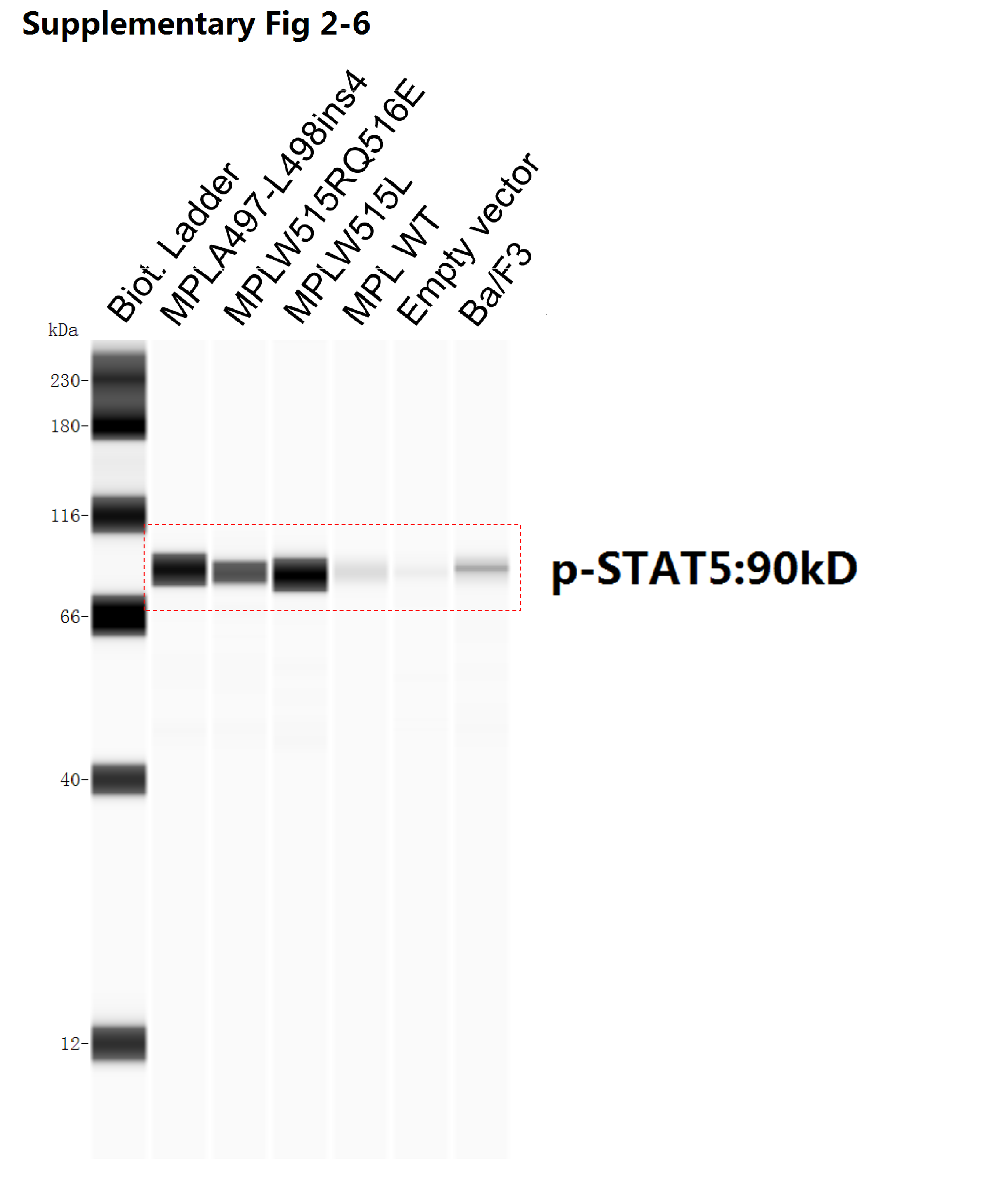

Supplement: Supplementary file 1 [file CAM4-8-5254-s001.zip › 6.p-stat5.tif]

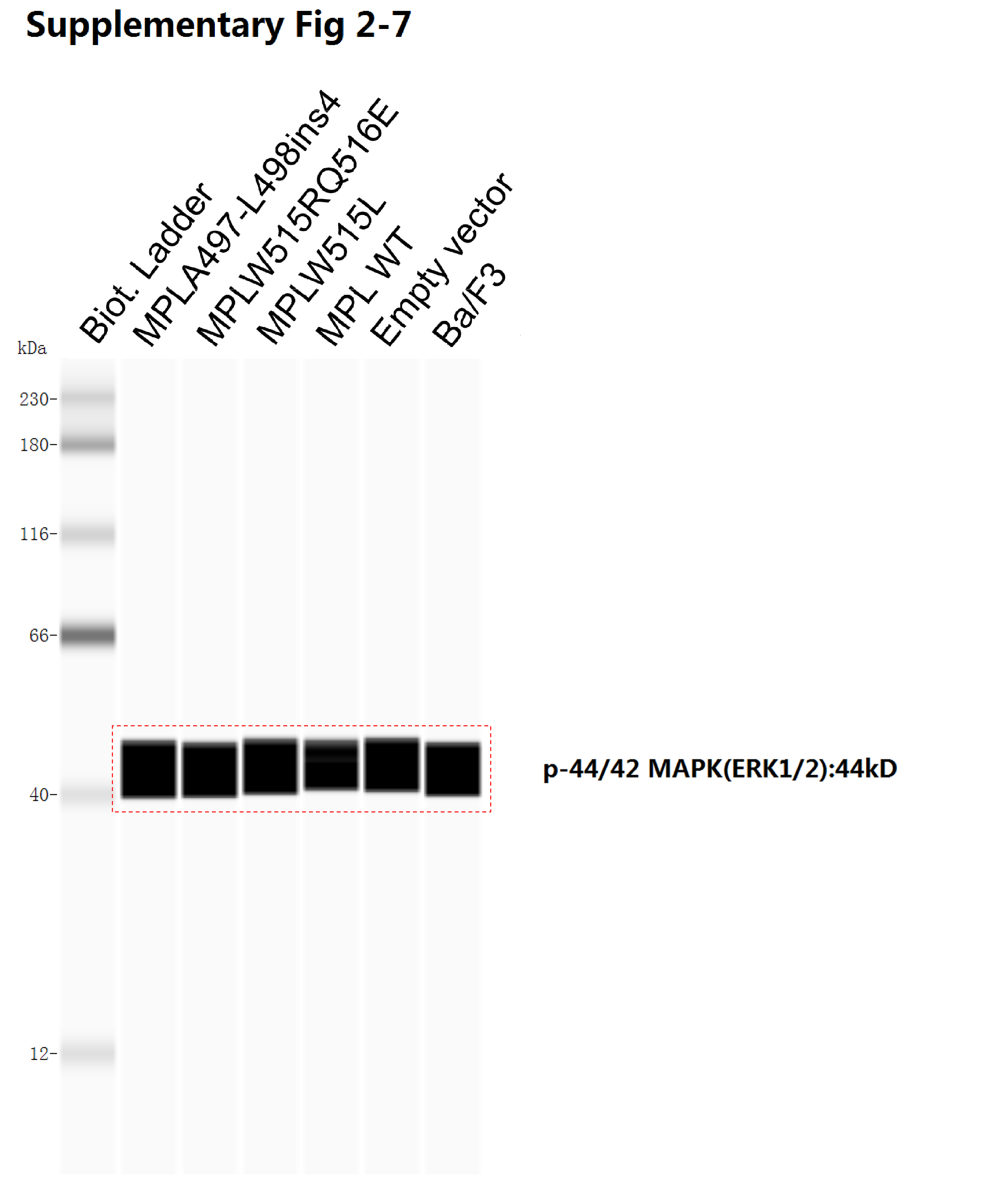

Supplement: Supplementary file 1 [file CAM4-8-5254-s001.zip › 7.erk.tif]

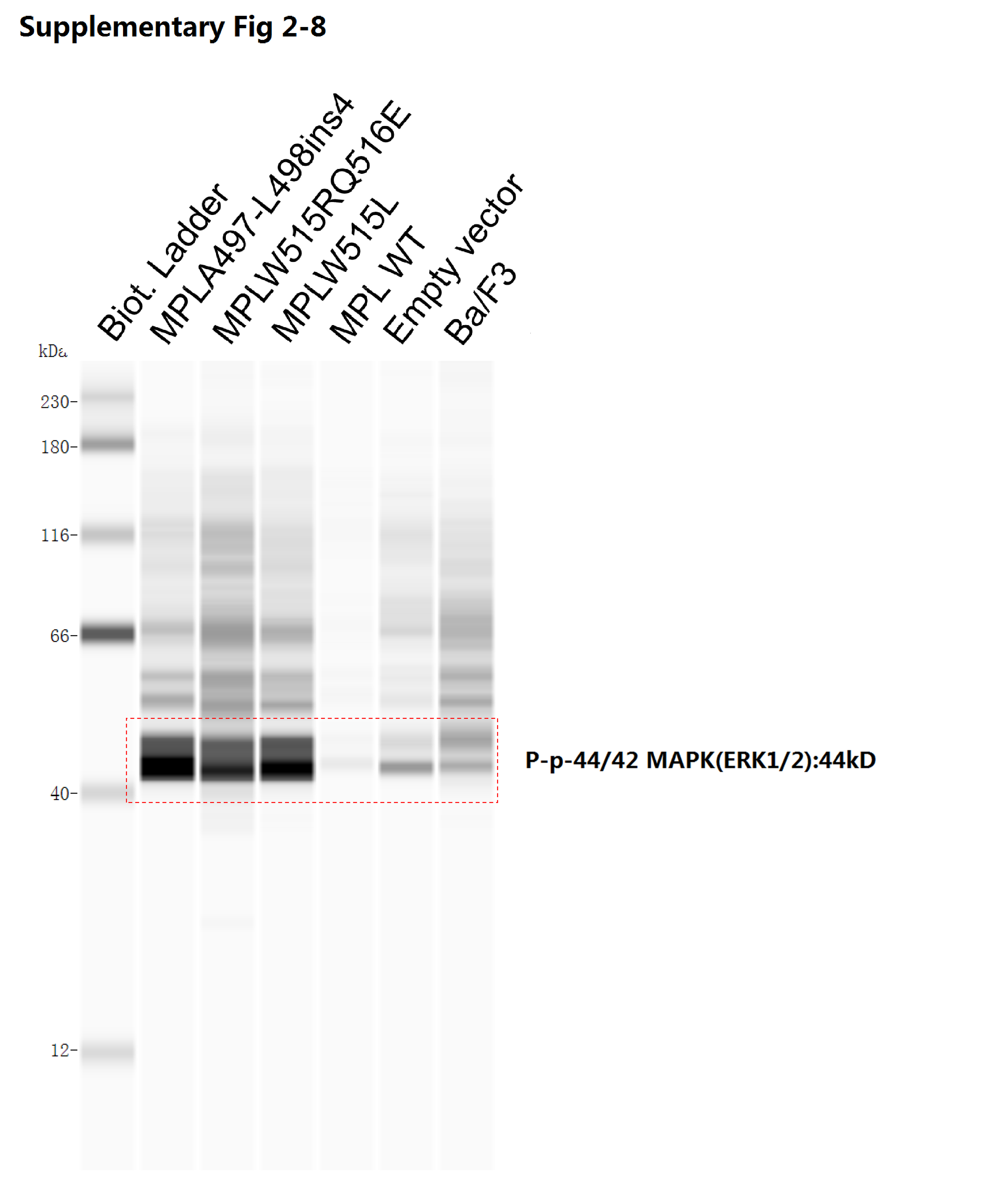

Supplement: Supplementary file 1 [file CAM4-8-5254-s001.zip › 8.p-erk.tif]

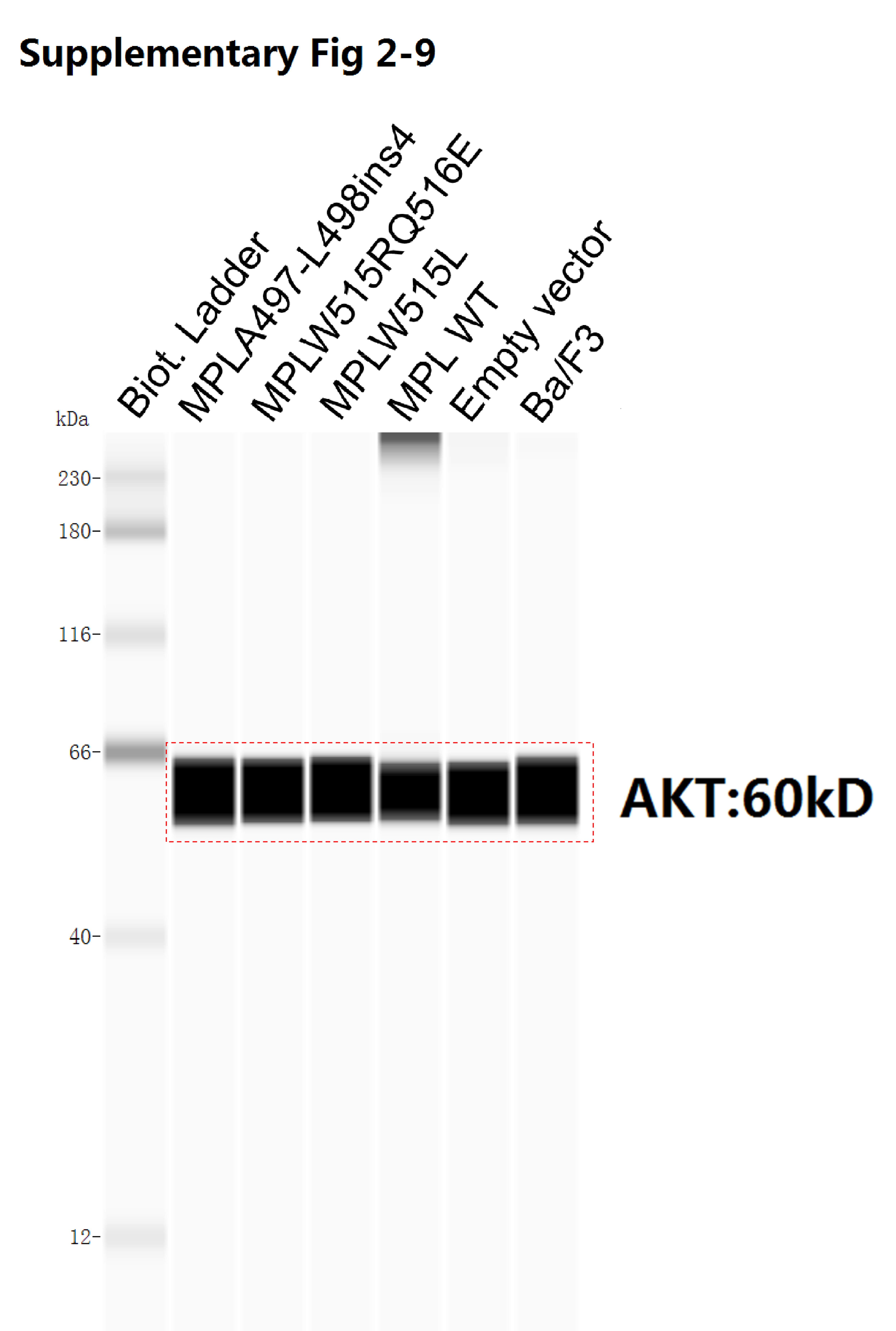

Supplement: Supplementary file 1 [file CAM4-8-5254-s001.zip › 9.AKT.tif]
